# Bowhead whales use two foraging strategies in response to fine-scale differences in zooplankton vertical distribution

**DOI:** 10.1038/s41598-020-76071-9

**Published:** 2020-11-20

**Authors:** Sarah M. E. Fortune, Steven H. Ferguson, Andrew W. Trites, Justine M. Hudson, Mark F. Baumgartner

**Affiliations:** 1 Marine Mammal Research Unit, Institute for the Oceans and Fisheries, University of British Columbia, Vancouver, BC V6T 1Z4 Canada; 2 Present Address: Fisheries and Oceans Canada, Freshwater Institute, Winnipeg, MB R3T 2N2 Canada; 3 Biology Department, Woods Hole Oceanographic Institution, Woods Hole, MA 02543-1050 USA

**Keywords:** Ecology, Ocean sciences

## Abstract

As zooplanktivorous predators, bowhead whales (*Balaena mysticetus*) must routinely locate patches of prey that are energy-rich enough to meet their metabolic needs. However, little is known about how the quality and quantity of prey might influence their feeding behaviours. We addressed this question using a new approach that included: (1) multi-scale biologging and unmanned aerial system observations of bowhead whales in Cumberland Sound, Nunavut (Canada), and (2) an optical plankton counter (OPC) and net collections to identify and enumerate copepod prey species through the water column. The OPC data revealed two prey layers comprised almost exclusively of lipid-rich calanoid copepods. The deep layer contained fewer, but larger, particles (10% greater overall biomass) than the shallow prey layer. Dive data indicated that the whales conducted long deep Square-shaped dives (80% of dives; averaging depth of 260.4 m) and short shallow Square-shaped dives (16%; averaging depth of 22.5 m) to feed. The whales tended to dive proportionally more to the greater biomass of zooplankton that occurred at depth. Combining behavioural recordings with prey sampling showed a more complex feeding ecology than previously understood, and provides a means to evaluate the energetic balance of individuals under current environmental conditions.

## Introduction

Bowhead whales (*Balaena mysticetus*) feed on patchily distributed prey such as amphipods, copepods, cirripedes, gastropods, euphausiids and mysids in the eastern Arctic^1–3^. Like other large, zooplanktivorous predators, they must consistently locate energy-rich prey patches (e.g., North Atlantic right whales^4–6^), which are in turn controlled by temperature, salinity, ice-formation and recession, phytoplankton availability, and mixing^7,8^. However, the predictability with which important prey such as calanoid copepods occur is likely to change in the North Atlantic and Arctic Ocean^9–14^ due to rapid changes in sea surface temperature and ice conditions^15–17^.

Understanding the implications of future shifts in zooplankton species composition, abundance and distribution on bowhead whales requires knowing what foraging strategies they employ and what they eat under present environmental conditions. However, relatively little is known about the feeding behaviour and primary prey of bowhead whales throughout their range—particularly in Canadian waters. What is known about bowhead diet in the Eastern Canadian Arctic has come qualitatively from stomach content analysis from a few harvested animals^2^, or has been inferred from stable-isotope^3,18^ and fatty acid^19^ analysis. Eastern Canada-West Greenland (ECWG) bowhead whale diet and behaviour has only been well studied in the eastern limit of their range in Disko Bay (western Greenland).

Disko Bay is predominately occupied by adult female ECWG bowhead whales during late winter and early spring for feeding^1,20,21^. In this area, bowheads are known to feed at depth primarily on a temperate/subarctic calanoid copepod, *Calanus finmarchicus*^1^. This is consistent with expectations of zooplankton species composition in Disko Bay based on the prevalence of the Western Greenland Current that contains both North Atlantic and Arctic water masses^21,22^. However, it is not known what prey juvenile and adult male whales consume because they are seldom seen in Disko Bay. It is also unknown what prey the Disko Bay females consume at other times of year, such as summer when they are elsewhere within their ECWG range and peak feeding is thought to occur^23^, and what feeding strategies they use.

Cumberland Sound, Nunavut (Canada) is another important area for ECWG bowhead whales. The abundance of whales in Cumberland Sound has been higher than any other region in the eastern Canadian Arctic (~ 20–40% of total population) based on analysis of aerial survey data collected in 2013 and skin biopsies used for genetic analysis obtained between 1995 and 2013^24–26^. Unlike Disko Bay, roughly equal numbers of male and female whales use Cumberland Sound^25^, and both juvenile and adult animals occupy this habitat^20^. The species composition of zooplankton in Cumberland Sound is likely to differ from Disko Bay and may be dominated by large-bodied, energy rich Arctic taxa such as *Calanus hyperboreus* and *C. glacialis*^27–29^ due to regional differences in ocean currents and water temperatures^22,30,31^. As a consequence, the feeding conditions may be improved in Cumberland Sound because of the availability of higher-energy prey (e.g., *Calanus hyperboreus* and *C. glacialis*^32,33^). Such an energy differece between feeding habitats may mean that Cumberland Sound is better able to support the comparatively high energy needs of juvenile and lactating females^6^ more readily than if they fed in Disko Bay.

The goal of our study was to evaluate the relationship between the vertical distribution of zooplankton and the fine-scale foraging behaviour of bowhead whales in Cumberland Sound and to explain why they primarily use one fiord within the region and exhibit bimodal dive behaviour. A second objective was to identify diet composition and evaluate the importance of Cumberland Sound as a foraging ground under current environmental conditions. We therefore collected: (1) fine-scale bowhead whale dive behaviour (time-depth recorders; hours); (2) long-term vertical and horizontal movement (time-depth recorder telemetry tags; days); and (3) surface behaviour (unmanned aerial system-UAS; minutes). We then correlated bowhead behavioural data with information about the species composition, abundance and vertical distribution of their prey by determining: (4) vertical particle size and abundance (optical plankton counter), (5) calanoid copepod species composition (integrated water column net tows); and (6) bowhead whale diet (stomach contents). Combined, these data are important for understanding how the ECWG population forages under present oceanographic conditions in the eastern Canadian Arctic.

## Results

### Fine-scale bowhead dive behaviour

**Figure 1:**
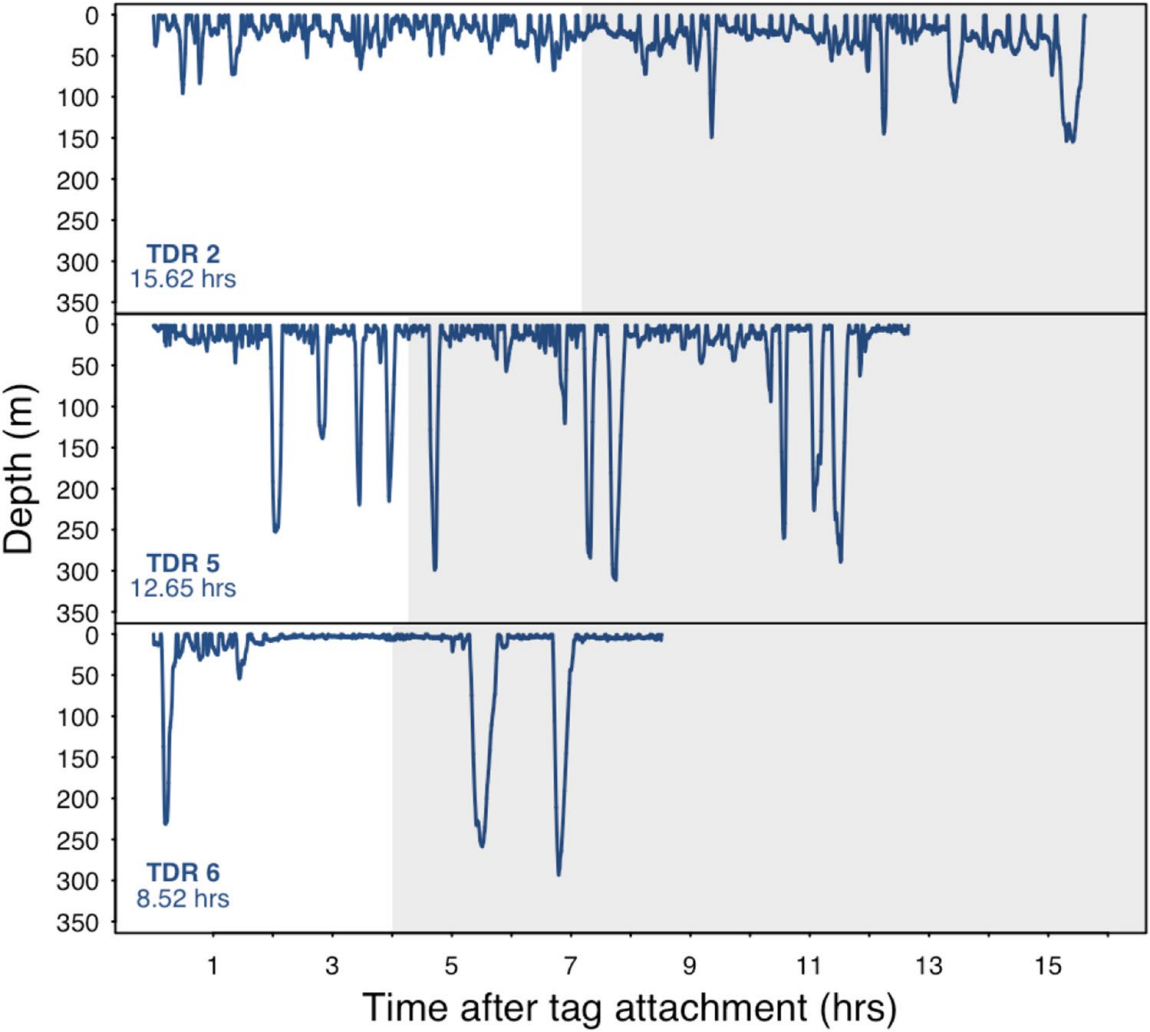
Time-depth recorder data (1 Hz sampling frequency) for 3 bowhead whales showing day and night-time behaviour (between sunset and sunrise—shaded grey where sunset occurred at 23:57 GMT). TDR 2 was tagged on 26 August 2016, and TDR 5 and TDR 6 were tagged on 29 August 2016.

The 6 bowhead whales equipped with fine-scale TDR tags had attachment times ranging from 0.8 to 15.6 h (Table S1). To obtain undisturbed dive behaviour, we subsequently left three animals after tagging (i.e., did not conduct a focal follow post tagging) and we obtained 8.5–15.6 h of diving behavioural data including dives that occurred during day and night (Fig. 1). Unlike the dive data for the focal followed animals, we found that the non-focal followed whales dove to various depths. The deepest and longest dives were between 115.1 and 305.0 m (220.6 m ± 63.2 SD) and lasted for 7.6 to 27.6 min (14.2 min ± 6.2 SD). In total, the tagged whales made 170 dives below 10 m and the majority were Square-shaped (46%) followed by U-shaped (27%) and V-shaped (27%). The Square-shaped dives were generally shallow but variable in depth, occurring between 10.1 and 249.6 m (25.7 m ± 28.1 SD). In comparison, U (70.7 m ± 88.5 SD) and V (60.1 m ± 65.9 SD) shapes dives were deeper occurring between 10.0–305.0 m and 10.5–284.1 m respectively.

### Telemetry

Eleven bowheads were equipped with Wildlife Computers SPLASH tags in Cumberland Sound between 20 and 28 August 2016, with usable data obtained from 9 tags (Table S2). Of those 9 tagged animals, 3 were male and 6 were female (5 males and 6 females tagged in total), with body sizes ranging from 8.5 to 11 m based on visual estimates of body length (distance between tip of whale’s snout and fluke notch) from the vessel. These estimates suggested that the tagged animals were young juveniles (1–4 years) and sexually immature sub-adults (> 4 years and < 25 years)^34–36^.

### Horizontal movement

**Figure 2:**
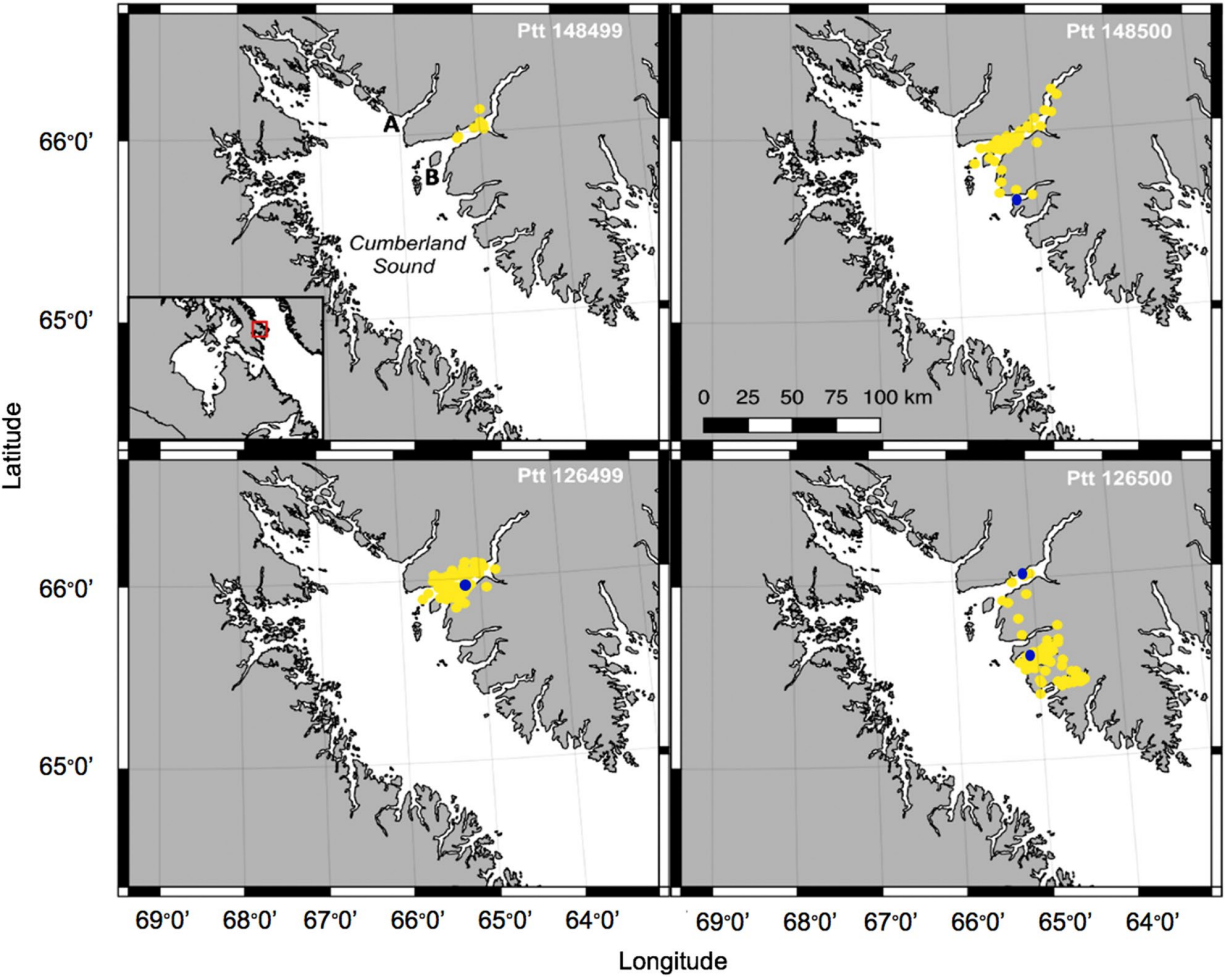
Locations (2 per day) of 4 bowhead whales (Ptt 126499, 126500, 148499, 148500) tagged with SPLASH tags in Kingnait Fiord during August 2016. Locations were predicted using a hierarchical switching state-space model (HSSSM), and were categorized by behavioural states—area-restricted movement (i.e., probable feeding) (yellow dots) and an unknown behavioural state (blue dots). Inset with red rectangle highlights study region—Cumberland Sound, Nunavut (Canada). Pangnirtung Fiord (**A**) and Kingnait Fiord (**B**) are labeled for identification purposes. The maps were made using QGIS 2.18 (https://qgis.org/en/site/forusers/download.html).

We constrained our analysis to telemetry data collected in Cumberland Sound during August and September 2016 to permit comparison with collected prey data. Two locations were predicted per day for each animal using the hierarchical switching-state-space model (HSSSM) resulting in 454 locations in total. The tags transmitted for 26 ± 10 SD days in Cumberland Sound on average with a total of 50 ± 22 SD predicted locations on average during August and September (range: 7–76 days). During August, we obtained 6 ± 3 SD days (range: 3–12 days) of movement data and 20 ± 9 SD days for September on average (range: 4–30 days). Five animals spent all of their time during August and September in Kingnait Fiord based on the HSSSM (n = 251 ARGOS locations; n = 5 animals). This represents 55% of all predicted locations (n = 454 ARGOS locations; n = 9 animals). Three animals spent most of their time in Kingnait Fiord and another left Kingnait Fiord shortly after being tagged and resided in adjacent fiords (e.g., Ptt 126500; Fig. 2). From an analysis of the HSSSM behavioural states 98% were consistent with area restricted movement (ARM) and 2% were of an unknown behavioural state. The dominance of ARM behaviour suggests that the tagged bowheads engaged in feeding-related activities daily (Fig. 2).

### Vertical movement

The 9 tagged bowhead whales dove a total of 5981 times (using > 10 m dive definition) over 41 days in Cumberland Sound (including fiords) during the day and night in August and September 2016 after excluding the data from the day the animal was tagged. The whales conducted predominately 77.1% Square (n = 4613) and 18.9% U-shaped dives (n = 1133), whereas V-shaped dives represented only 3.93% (n = 235) of the total. We inspected the summary dive statistics (e.g., range, mean ± SD) for unusually high values that would exceed the physiological diving limits of the species and found no biologically improbable dives that exceeded 75 min in duration or 700 m in depth.

**Figure 3:**
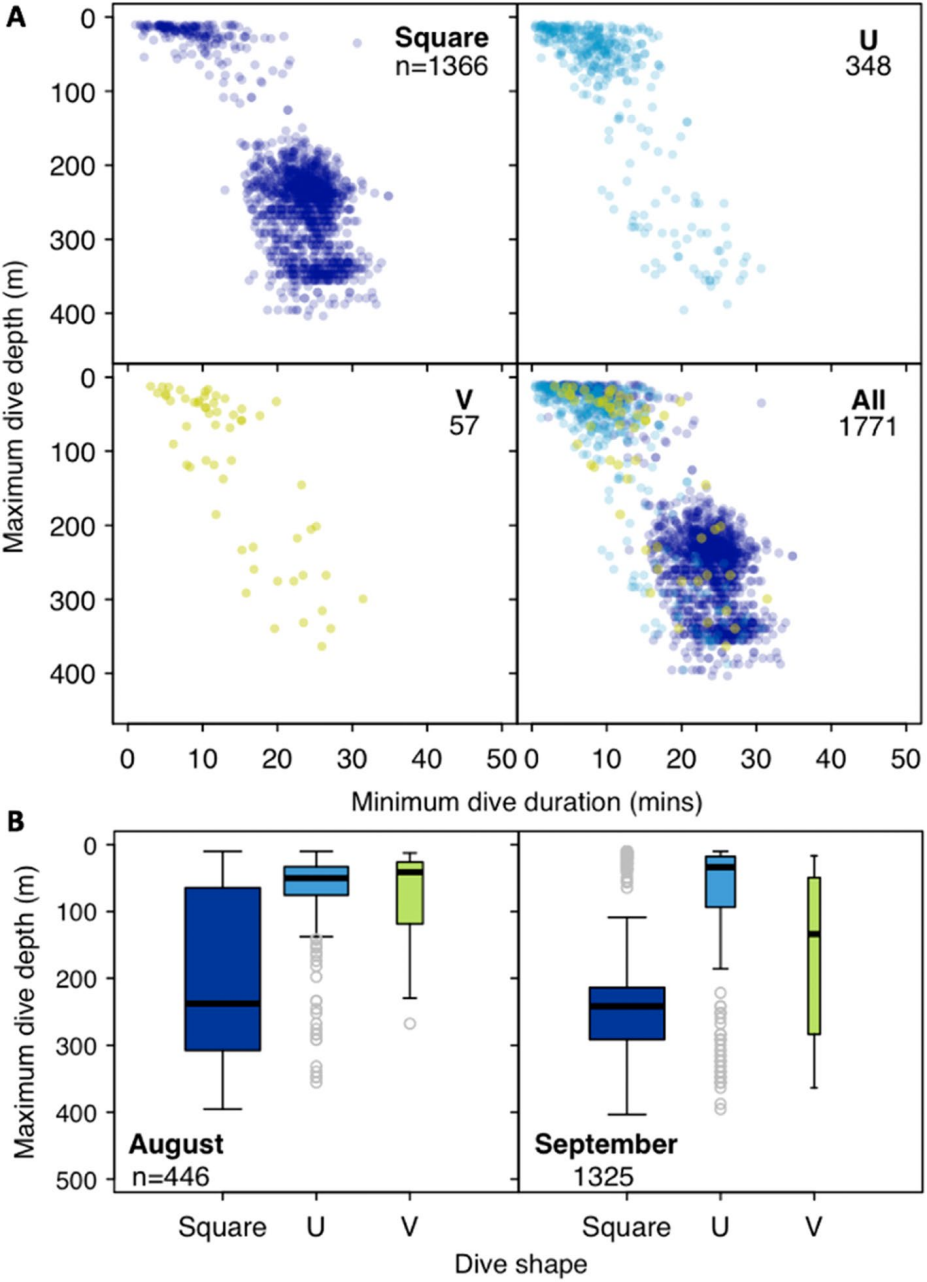
(**A**) All daytime dives for 5 SPLASH tagged bowhead whales (Ptt 126499, 148499, 148502, 148504, 148505) that resided in Kingnait Fiord during August and September 2016. Dive types are differentiated in the panels by colour (Square: dark blue; U: light blue; V: green). (**B**) daytime diving depths by shape of the dives (Square, U and V) made by 4 SPLASH tagged bowhead whales that were in Kingnait Fiord during August (n = 446 dives) and 5 during September 2016 (n = 1325 dives). The width of each boxplot is proportional to the square root of the sample size. There were n = 241 Square dives, n = 176 U-shaped dives and n = 29 V-shaped dives during August, and n = 1125 Square dives, n = 172 U-shaped and n = 28 V-shaped dives during September inside Kingnait Fiord.

We found that 56.4% (n = 833 day only dives; total dives n = 1477 day and night) and 51.0% (n = 2297 day only dives; n = 4504 day and night) of all classified dives occurred during the day in August and September, respectively (Table S3). The tagged whales conducted proportionally more Square-shaped dives than U- or V-shaped dives during August (64.5%; total classified daytime dives n = 833) and September (83.2%; n = 2297). Furthermore, we found that Square shaped dives were consistently deeper and longer in duration than any other dive shape (Fig. 3; Table S3). The whales dove to comparable mean depths during September (215.5 m ± 38.76 SD) and August (214.5 m ± 28.73 SD), but stayed longer at depth during September (September 21.3 min ± 2.61 SD; August 18.4 ± 1.52 SD) based on weighted averages (Fig. 3). However, during August, 16.39% (total Square dives n = 537) of all Square-shaped dives occurred in the top 50 m of the water column at 22.48 m ± 4.51 SD and 79.9% (n = 429) occurred below 100 m at an average of 260.42 m ± 35.83 SD suggesting that the whales alternate between shallow and deep feeding dives. Furthermore, U-shaped dives consistently occurred at a similarly shallow depth during August (72.41 ± 29.03 SD) and September (84.37 ± 18.66) based on a weighted average (Table S3). Consequently, we found that dive duration (Likelihood Ratio Test (LRT) = 1217.2, p < 0.0001; Model 3; Table S4) varied by shape and month, while maximum dive depth varied by dive shape (LRT = 0.422.5, p < 0.0001; Model 4; Table S4) but not by month (LRT = 0.169, p = 0.6809; Model 5; Table S4).

We examined the Square and U-shaped dive depth of SPLASH tagged whales in Cumberland Sound during the day and night and found no statistical evidence of diel diving behaviour (Fig. S1; Table S4). For example, in August during the day, the average depth of square dives was .5 m (± 110.1 SD), which was similar to the mean dive depth at night 222.3 (± 123.7 SD). U-shaped dives tended to be shallower, but occurred at comparable depths during the day (73.3 ± 82.3 SD) and night (70.3 m ± 78.9 SD) in August. Consequently, the maximum depth of dives differed by shape (Square, U, V-and Unknown) (LRT = 210.5, p < 0.0001; Model 6) but not by periods of daylight (day) and darkness (night) (LRT = 0.396, p = 0.5293; Model 7).

We found that 4 out of the 9 tagged animals spent their time (2–8 days) exclusively in Kingnait Fiord during August, and the remaining 5 animals resided exclusively (3–30 days) in Kingnait Fiord during September based on the predicted HSSSM locations. While in Kingnait Fiord, the whales conducted greater numbers of deeper dives (63% ≥ 100 m; n = 202 dives) than shallow dives (37% ≤ 50 m; n = 121). For example, individuals alternated between deep (≥ 100 m) Square (267.4 m ± 29.15 SD; n = 170 dives) and U-shaped (242.7 m ± 25.82 SD; n = 32 dives) dives and shallow (≤ 50 m) Square and U-shaped dives that occurred at 24.67 m ± 2.06 SD (n = 32 dives) and 28.9 m ± 3.22 SD (n = 89 dives), respectively, on average (Fig. 3).

### Unmanned aerial systems behavioural observations

During 2015, we obtained high-resolution aerial images (n = 1143) and video of tagged and untagged bowhead whales using a small unmanned aerial system (UAS), the DJI Phantom 3 Professional. On average, the UAS was flown at an altitude of 12.9 m (± 5.4 SD) and was within 1000 m of the research vessel with flight times between 8 and 12 min.

Conducting focal follows of tagged animals with the UAS revealed that bowhead whale behaviour changed in response to our vessel. During our focal follows, the whales would often travel towards the shoreline and would spend considerable time at the surface. We observed this behavioural response with and without the use of an UAS. Examination of the fine-scale dive data for the focal followed animals showed that the whales were conducting principally short and shallow dives immediately following tagging. Furthermore, we rarely recorded near-surface feeding events. We obtained still images with animals that had slightly agape mouths, however, they were occupying shallow, coastal waters where prior prey sampling revealed extremely low abundances of zooplankton. Consequently, the drone confirmed that whales were not engaged in feeding activities while we conducted focal follows suggesting that visual observations may underestimate actual foraging activity and/or few feeding activities occurred while we conducted focal follows.

### Optical plankton counter particle size, abundance and biomass

**Figure 4:**
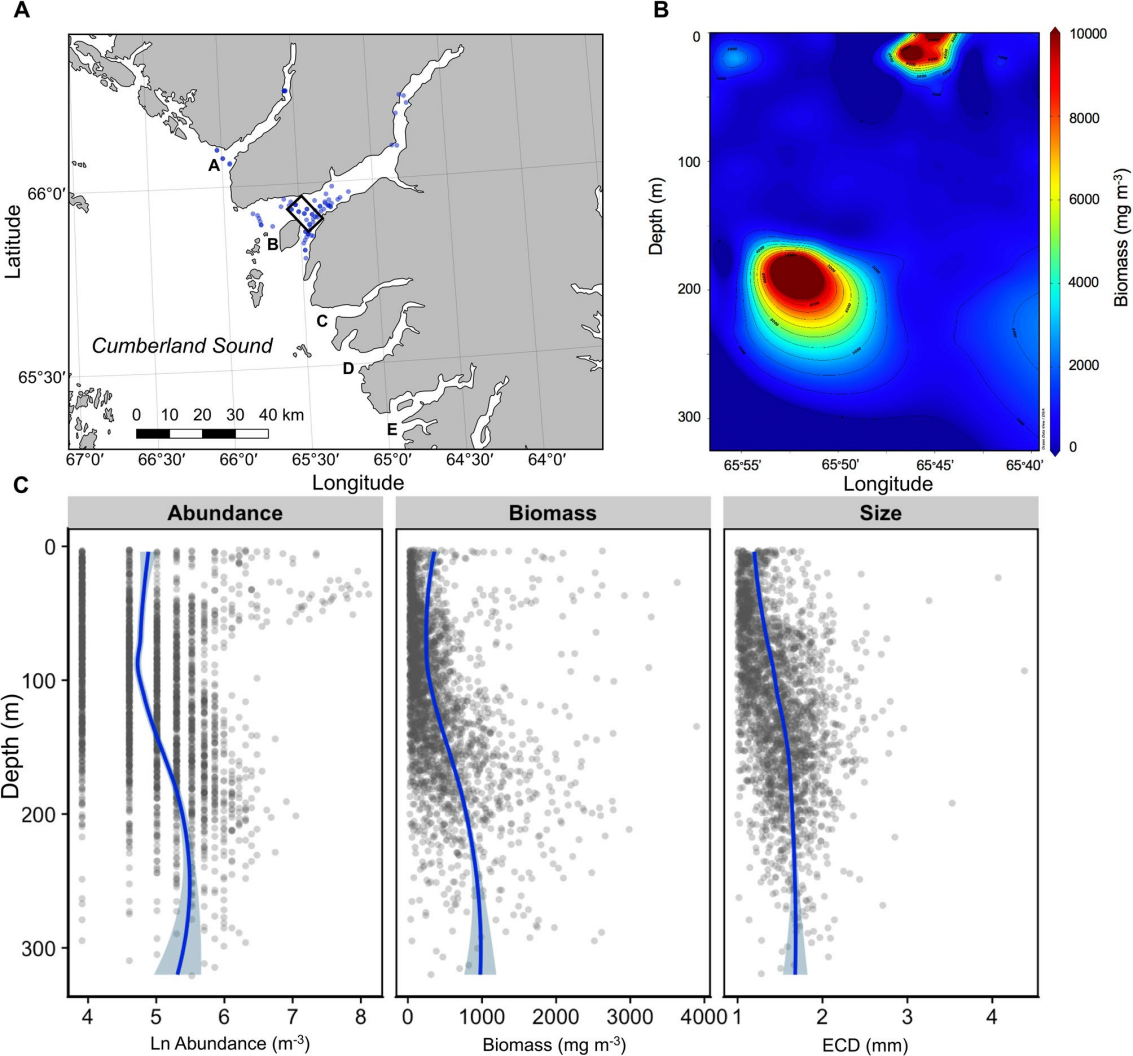
(**A**) Locations of all OPC vertical casts (blue circles) in 2016, and a sub-set of the casts (n = 16) (black box) across Kingnait Fiord behind Kekertukdjuak Wesland where bowhead whales made long, high-fluking dives and were presumed to feed. For reference, we identified fiords of interest where: A = Pangnirtung Fiord, B = Kingnait Fiord, C = Weqalujjaq Fiord, D = Unnamed, E = Kumlien Fiord and F = Ujuktuk Fiord. The map was made using QGIS 2.18 (https://qgis.org/en/site/forusers/download.html). (**B**) Vertical particle biomass concentrations within the sub-set area were particularly high (≥ 1000 particles/m^−3^) for some of the OPC casts at the surface and at depth. This figure was generated using Ocean Data View 5.2.0 (https://odv.awi.de) copy right 2019 Reiner Schlitzer. (**C**) OPC data for 72 casts made in Kingnait Fiord that were sorted into 4 m depth bins by particle Ln abundance (m^−3^), biomass (mg m^−3^) and mean equivalent circular diameter (mm) (e.g., particle size). To minimize data overlap, the data were jittered and a loess curve (i.e., locally estimated scatterplot smoothing) was fitted to the data to assist in data trend visualization.

Of the 72 vertical OPC casts in Kingnait Fiord, 52 were made in association with bowhead whales. The depth stratum with the highest abundances of particles ≥ 1.0 mm (2146.9 particles m^−3^ ± 778.3 SD; range = 1050–3950 particles m^−3^) was between 30 and 40 m (n = 16). We detected two possible prey layers (based on high particle abundance)—a shallow (5–55 m) and deep (190–225 m) layer. Particle abundances were highly variable in the shallow layer and ranged from 50 to 3,950 particles m^-3^ (mean particle abundance: 294.1 ± 542.2 SD) compared with abundances at depth that varied from 50 to 1,150 particles m^-3^ (mean particle abundance: 285.4 ± 175.4 SD) (Fig. 4). We found that mean equivalent circular diameter (ECD) was 1.46 ± 0.53 SD when averaged across the entire water column and that particle size increased with increasing depth. Particle sizes were 25% larger at depth whereby the ECD averaged 1.21 m ± 0.49 SD between 5 and 55 m and averaged 1.63 mm ± 0.52 SD between 190 and 225 m (Fig. 4). When particle sizes were converted to biomass (wet weight m^−3^) for each cast (Fig. 4), we found that estimated biomass was 10% higher and less variable on average in deeper layers (190–225 m; 979.39 mg m^−3^ ± 378.25 SD) compared with shallow layers (5–55 mm; 886.79 mg m^−3^ ± 2853.88 SD). Furthermore, when we plotted biomass concentration in an area where bowheads appeared to frequently engage in feeding behaviour (e.g., high fluking and long dives) during daytime in Kingnait Fiord behind Kekertukdjuak Wesland (Fig. 4), we found that particle biomass was similarly high near the surface (5–55 m) and comparatively higher at depth (190–225 m). High biomass appears to occur because of greater particle abundance (Fig. 4) in the surface and because of larger particles on average at depth (Fig. 4), which is consistent with what we observed elsewhere in Kingnait Fiord.

We obtained 11 vertical OPC casts in Pangnirtung Fiord where the maximum sampling depth ranged from 52 to 112 m. We found that particle abundances ≥ 1000 particles m^-3^ occurred between 8 and 36 m (25.7 m ± 12.41 SD) (n = 4 casts and n = 7 layers) and ranged from 1000 to 3000 particles m^−3^ (1607.1 particles m^−3^ ± 707.9 SD). Only one prey layer was detected, and it occurred between 5 and 40 m (325 particles m^−3^ ± 458.3 SD; range: 50–3000 particles m^3^). Particle abundances were considerably less at depth (90–110 m) and ranged from 50 to 250 particles m^−3^ (106.3 particles m^−3^ ± 72.89 SD). The average integrated water column ECD was 1.33 mm ± 0.41SD and particle size increased with increasing depth whereby the average ECD for particles between 5–40 m was 1.26 mm ± 0.36 SD and 1.38 mm ± 0.34 SD between 90 and 110 m. Unlike Kingnait Fiord, we found that estimated biomass was 45% greater at the surface (527.02 mg m^−3^ ± 404.25 SD; 5–40 m; n = 11) than at depth (291.82 mg m^−3^; 90–110 m; n = 1).

### Temperature and salinity profiles

**Figure 5:**
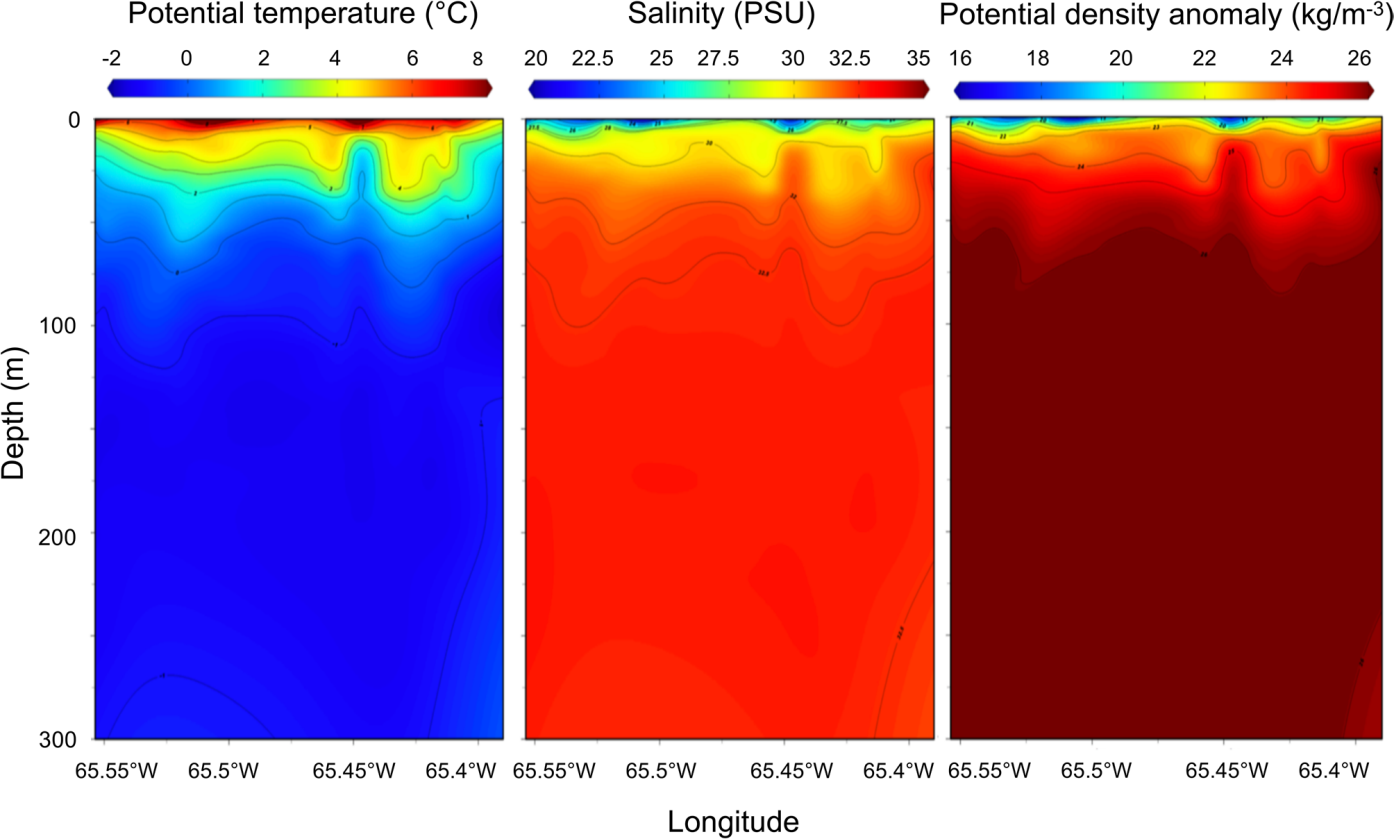
Potential temperature (°C) salinity (PSU) and potential density anomaly (kg m^−3^) contour plots for n = 16 CTD profiles made across Kingnait Fiord behind Kekertukdjuak Wesland where bowhead whales made long, high-fluking dives and were presumed to feed. This figure was generated using Ocean Data View 5.2.0 (https://odv.awi.de) copy right 2019 Reiner Schlitzer.

The 72 co-located CTD and OPC casts in Kingnait Fiord during August 2016 revealed strong water column stratification, consistent with sub-Arctic fiords during summer whereby the surface (1 m) or local water (i.e., freshwater inputs from rivers) was considerably fresher (26.84 PSU ± 2.28 SD) and warmer (6.57 °C ± 1.20 SD) compared with the intermediate (100 and 200 m) or advected coastal water (i.e., originating from Cumberland Sound and Davis Strait) that was considerably cooler (− 0.83 °C ± 0.75 SD at 100 m and − 1.25 °C ± 0.56 SD at 200 m) and higher in salinity (32.41 PSU ± 1.23 SD at 100 m and 32.26 PSU ± 1.81 SD at 200 m). The salinity gradient (n = 16) in the middle of Kingnait Fiord where bowheads appeared to feed during daytime was somewhat stronger and more abrupt than the temperature gradient (which showed more gradual changes with increasing depth) (Fig. 5). This suggests that vertical differences in salinity may have driven the apparent vertical density differences (Fig. 5) in the top 50 m of the water column. Furthermore, we found that the average base depth of the mixed layer occurred at 17.06 m ± 9.09 SD for CTD casts made in the middle of Kingnait Fiord.

### Zooplankton net sample abundance and biomass

**Figure 6:**
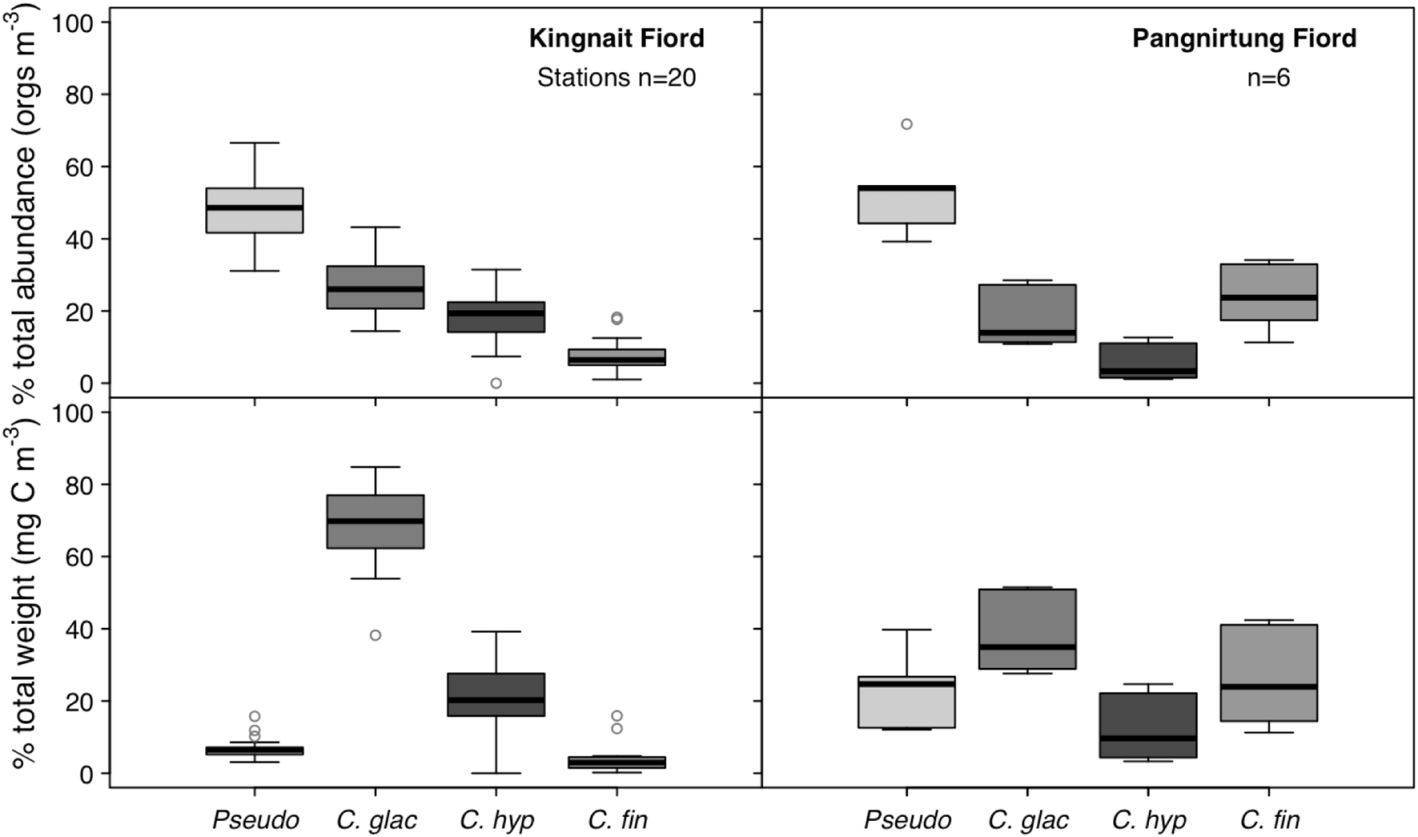
Proportion of total abundance (top) and proportion of estimated dry weight (bottom) of most common individual calanoid copepods (4 taxa) sampled from 6 net tows in Pangnirtung Fiord and from 20 tows in Kingnait Fiord. For zooplankton abundances, taxa included *Pseudocalanus* spp., *Calanus finmarchicus*, *C. glacialis*, *C. hyperboreus*, *Metridia* spp., *Oithona* spp. and *Acartia longiremis*. For dry weights, taxa included *Calanus glacialis* (n = 863), *C*. *hyperboreus* (n = 1754), *C. finmarchicus* (n = 1135) and *Pseudocalanus* spp.^37^. The boxplots are shaded according to the relative total caloric content per individual of each taxa from lowest energy prey (*Pseudocalanus* spp.) to greatest energy content (*C. hyperboreus*)^32,38^.

The 26 zooplankton net samples collected in Pangnirtung (n = 6) and Kingnait Fiord (n = 20) between 5 and 28 August 2016 (Tables S5–S10) contained an average of 91% (± 8.8 SD) calanoid copepods of which 48% (± 11.4 SD) consisted of *Calanus* spp. Of these Calanidae *Calanus* spp. 78% (± 19.7 SD) were Arctic taxa (e.g., *Calanus hyperboreus* and *C. glacialis*) and 22% (± 19.7 SD) were of temperate/subarctic origin (e.g., *Calanus finmarchicus*). Other taxa such as *Metridia lucens, M. longa, Acartia* spp. and *Oithona* spp. represented less than 5% of the enumerated organisms and were excluded from our biomass calculations due to their relatively small contribution to zooplankton assemblages in both Kingnait and Pangnirtung Fiord. When net samples were converted into abundance (orgs m^−3^) for the most common taxa, we found that *Pseudocalanus* spp. was similarly the most numerous taxa in Pangnirtung (52.98% ± 11.15 SD; 42.33 orgs m^−3^ ± 14.91 SD) and Kingnait Fiord (48.23% ± 10.21 SD; 13.98 orgs m^−3^ ± 4.45 SD). However, the second most abundant species were *C. finmarchicus* (23.86% ± 9.16 SD; 19.88 orgs m^−3^ ± 11.42 SD) in Pangnirtung Fiord and *C. glacialis* (26.14% ± 8.13 SD; 8.06 orgs m^−3^ ± 4.19 SD) in Kingnait Fiord (Fig. 6; Table S7).

Using our prosome measurements and previously established species-specific size ranges for *Calanus* spp.^39^, we found that all *Calanus* spp. were dominated by early copepodid stages (CI-CIV) (Fig. S2) and that this was particularly true for *Calanus finmarchicus* (96.1% ± 6.5 early stage). Numerically, *Pseudocalanus* spp. was the most abundant taxa in Pangnirtung and Kingnait Fiord representing 47% (± 9.3 SD) of all calanoid copepods, followed by *Calanus glacialis* (23.0% ± 8.9 SD), *C. hyperboreus* (14.3% ± 8.0 SD) and *C. finmarchicus* (10.8% ± 9.2 SD) (Fig. 6).

When we compared the copepod community composition between fiords (Fig. 6), we found that Pangnirtung Fiord (n = 6) contained a higher proportion of *Pseudocalanus* spp. 52.3% (± 11.2 SD) compared with Kingnait Fiord (n = 20) 48.2% (± 10.0 SD). We also found that *C. finmarchicus* was more dominant in Pangnirtung Fiord (23.9% ± 9.2 SD) than Kingnait Fiord (7.4% ± 4.8 SD) and that *C. glacialis* (26.1% ± 8.1 SD) and *C. hyperboreus* (18.2% ± 7.0 SD) were more dominant in Kingnait Fiord compared with Pangnirtung Fiord (*C. glacialis:* 17.7% ± 8.1 SD and *C. hyperboreus:* 5.5% ± 5.1 SD) (Fig. 6). However, when converted to dry weight (mg C m^−3^), we found that *C. glacialis—*an Arctic taxa—comprised the greatest proportion of total *Calanus* spp. and *Pseudocalanus* spp. biomass in Kingnait Fiord (Fig. 6).

In terms of biomass, 4359 *Calanus* spp. organisms were staged during enumeration and identification (n = 26 stations) and 86% had undamaged exoskeletons permitting prosome measurement (i.e., n = 3752) (Fig. S2). Of those measured, biomass was calculated using previously established allometric relationships between prosome length (μm) and dry weight (mg C). Unlike total numerical abundance, we found that zooplankton biomass was dominated by *Calanus glacialis* (68.6% ± 10.8 SD in Kingnait Fiord and 39.9% ± 9.61 SD in Pangnirtung Fiord) and *C. hyperboreus* (20.8% ± 9.3 SD in Kingnait Fiord and 12.3% ± 9.7 SD in Pangnirtung Fiord). This was particularly true for Kingnait Fiord (Fig. 6, Tables S7 and S8).

### Species composition of bowhead whale stomach sample

The sub-adult female bowhead whale harvested in Kingnait Fiord (65° 48′ 23″ N and 65° 28′ 12″ W) on 14 September 2016 measured 11.76 m—straight line distance from the tip of the snout to the fluke notch. The 500 mL sub-sample of stomach contents contained a total of 6 zooplankton taxa. Of the 477 enumerated organisms, 67.7% (n = 323) were *Calanus* spp., 18.7% (n = 89) were *Metridia* spp., 9.6% (n = 46) were unidentified copepods, 1.89% (n = 9) were *Pseudocalanus* spp., 1.89% (n = 9) were amphipods and 0.21% (n = 1) were mysids. We found that the *Calanus* species were dominated by early-stage (CI-CIV) *Calanus hyperboreus* (26.3%, n = 85) and late-stage (CV-Adult) *C. glacialis* (24.5%, n = 79) and *C. finmarchicus* (1.2%, n = 4) (Table S10).

## Discussion

Our analysis of multi-scale bowhead whale diving behaviour and fine-scale zooplankton distribution and abundance provides new insights into the relationship between bowhead whale foraging behaviour and the quality and quantity of prey throughout the water column. Most notably, we found that bowheads employed a more plastic feeding strategy than we expected. Whales appeared to exploit two discrete prey layers during the daytime—a shallow layer that consisted of higher abundances of smaller prey, and a deep layer that was of comparatively lower abundance but higher in biomass. Furthermore, we obtained new insight regarding the diet composition of bowhead whales whereby differences in zooplankton species composition between two adjacent fiords provided evidence that the whales principally occupied Kingnait Fiord because of the dominance of large, lipid rich Arctic copepods such as *Calanus glacialis.* These findings confirm that Cumberland Sound, Nunavut is a summertime feeding habitat for Eastern Canada-West Greenland bowhead whales and may be used to infer bowhead whale habitat preferences.

### Habitat use patterns

The discovery and exploitation of bowhead whales in Cumberland Sound during the mid-nineteenth century was essential to the resurgence of the commercial hunt after bowheads were overharvested in the high Arctic^40^. Scottish whalers established the Kekerten Wesland Whaling Station on Qikiqtaq Wesland near the mouth of Kingnait Fiord in 1857^41^. The station was strategically placed to allow the whalers to spot bowheads from shore using a telescope and to overwinter in preparation for the spring hunt^41^. The Kekerten Wesland Whaling Station became one of the most significant and longest operating whaling stations in Cumberland Sound—evidence that Kingnait Fiord is a historically important habitat for bowhead whales.

Eastern Canada-West Greenland bowhead whales continue to occupy Kingnait Fiord while their population recovers to pre-exploitation stock sizes^42^. Systematic boat-based surveys consistently found whales in this habitat compared with neighbouring fiords such as Pangnirtung Fiord during summer (July and August)^43,44^. Furthermore, our analysis of horizontal movement showed that bowheads have a high residency period in Kingnait Fiord, as over half of all predicted HSSSM locations occurred within this area during August and September. Five of the tagged animals remained exclusively within Kingnait Fiord during this time period. Almost all (98%) of the HSSSM locations were associated with area-restricted movement suggesting that the whales utilize Kingnait Fiord for feeding purposes. Although some animals made excursions to Weqalugaju Fiord (65° 38′ 58.6″ N 65° 17′ 10.6 W; a neighbouring fiord to Kingnait Fiord), no tagged animals appeared to occupy Pangnirtung Fiord. Consequently, it appears as though Kingnait Fiord continues to be an important summertime habitat for bowheads over other fiords in Cumberland Sound.

### Multi-depth feeding strategy

Bowheads exploited shallow and deep prey layers despite the deeper layer containing 46% more biomass. However, long-term SPLASH tag data showed that they dove proportionally more to depth—presumably to exploit deeper prey aggregations. For example, we found that while in Kingnait Fiord, SPLASH tagged animals partitioned their time between shallow Square (25 m) and U-shaped (30 m) dives and deep Square (267 m) and U-shaped ( 242 m) dives, which has been similarly observed for North Atlantic right whales in the Great South Channel^45^. The predominance of deep feeding dives recorded from SPLASH tagged animals is likely associated with the ontogenetic vertical migration of copepods, while the shallow dives may be focused on copepods that have not yet entered diapause (consistent with previous investigations of bowhead whale dive depth by month in Cumberland Sound^46^). Furthermore, we found that fine-scale tagged animals also alternated between shallow and deep probable foraging dives where several shorter, shallow dives typically followed single long deep dives. However, the proportion of deep feeding dives were considerably lower compared with the SPLASH tags. Differences in the proportion of time spent conducting deep foraging dives between the two tag types may be attributed to the comparatively smaller dataset using the fine-scale archival tags and the shorter duration of tag attachments (hours vs. days-weeks).

We also found that the mean dive depth (Fig. 3) coincided with the depths of maximum zooplankton abundance both at the surface (30–40 m) and at depth (190–225 m) (Fig. 4). The average maximum depth of deep probable foraging dives (260.42 m ± 35.83 SD) was somewhat deeper than the depth of the prey layers (190–225 m). Differences between the maximum dive depth and depth of maximum zooplankton biomass may be attributed to spatio-temporal differences in co-location between dive data and prey samples. For example, spatial variability in bathymetry^1^ may help explain this discrepancy such that the whales may have been conducting the majority of their dives in a deeper region of Kingnait Fiord than where the bulk of our prey samples were collected. Furthermore, the maximum depth of the dive may not correspond with the depth of actual prey ingestion as animals may adjust their depth during the bottom phase of their dive, as was observed during deep dives for animals equipped with the fine-scale TDR (Fig. 1).

Although the UAS was helpful at monitoring whale behaviour at the surface, observations of subsurface foraging activities were seldom made. Given the preponderance of probable foraging dives captured by both the long-term SPLASH tag and the short-term, fine-scale archival tag it suggests that visual observations of subsurface foraging behaviours are underestimated. The most likely explanations for this discrepancy are that: (1) the presence of phytoplankton and detritus limited the optical visibility of the water column preventing accurate detections of foraging at or below 30 m; (2) bowheads exhibit diel patterns in foraging behaviour such that shallow foraging occurs more frequently after dusk while deep foraging is most common during daytime; (3) it is also possible that the presence of the research vessel affected the behaviour of the animals during focal follows and biased visual observations of shallow feeding. These findings are an example of the inherent limitations of relying on visual observations alone to assess whale behaviour—one of the many benefits of using biologging technology^47–49^.

As in our study, bowheads were similarly found to alternate between a shallow and deep prey layer during the spring in Disko Bay (Western Greenland). Considerable variability in feeding dive depth (53–109 m)^1^ was observed whereby the whales appeared to exploit a prey layer between 30 and 60 m that had biomass dominated by Arctic taxa (*Calanus glacialis* and *C. hyperboreus*) and a deeper layer between 75 and 115 m that had biomass dominated by a temperate/subarctic species (*C. finmarchicus*). It was suggested that the deep layer included pre-ascension *C. finmarchicus* and that the shallower layer represented organisms that had ascended to support reproductive and feeding activities^1^*.* In our study, however, we found evidence of the reverse, whereby the deep layer likely represented descended Arctic taxa and the shallower layer likely included actively feeding temperate/subarctic species and non-overwintering life-stages of *C. glacialis* and *C. hyperboreus*. These differences in vertical distribution of *Calanus* spp. are consistent with what is known about seasonal ontogenetic movement^50–53^.

The dominance of Arctic copepods (e.g., *Calanus glacialis* and *C. hyperboreus*) relative to temperate/subarctic species suggests that prey quality may be higher in Cumberland Sound (summer sampling) than Disko Bay (late winter-early spring sampling). However, it is also possible that the high abundance of smaller-bodied temperate/subarctic species (e.g., *Calanus finmarchicus*) in Disko Bay^1,21^ is sufficient to outweigh the comparatively lower energy content. Due to the morphological similarity and size overlap between the three *Calanus* spp.^54^, we could not accurately convert the particle size measurements from the OPC into species and life-stage specific measurements. Consequently, future studies seeking to conduct quantitative comparisons of the prey quality between both habitats should collect prey samples using depth-stratified net or pump sampling methods to permit species identification and enumeration in particular prey layers.

### Zooplankton species composition

Bowhead whales likely select Kingnait Fiord over other areas in Cumberland Sound because of better feeding conditions. Zooplankton species composition from net collected samples revealed that smaller bodied taxa such as *Pseudocalanus spp.* (representing  48% and  53% of total abundance on average in Kingnait and Pangnirtung Fiord) and *Calanus finmarchicus* (24% total abundance in Pangnirtung Fiord) were considerably more abundant than Arctic *Calanus* spp. (Fig. 6 and Table S7). Differences in copepod assemblages translated into considerably lower biomass estimates (mg C m^−3^) for larger-bodied, higher energy Arctic species such as *Calanus glacialis* and *C. hyperboreus* in Pangnirtung Fiord (52.2% of total biomass per cubic meter on average) compared with Kingnait Fiord (89.4%) (Fig. 6 and Table S7). Arctic species of *Calanus* are higher in energy content (e.g., *Calanus glacialis* contains 0.38–0.45 mg of lipid per late-stage individual^32^) compared with temperate/sub-Arctic species (e.g., *Calanus finmarchicus* contains 0.04–0.08 mg of lipid per late-stage individual)^32^. Consequently, the quality of prey available to bowhead whales appears to be greater in Kingnait Fiord compared with Pangnirtung Fiord due to the presence of large-bodied and energy-rich Arctic taxa in Kingnait Fiord.

Biomass estimates from OPC data showed that the near surface prey layers were comparable between fiords with Kingnait Fiord having only marginally higher biomass than Pangnirtung Fiord. However, the absence of a deep layer that is consistently high in biomass in Pangnirtung Fiord may help explain why whales were more numerous in Kingnait Fiord. The absence of a deep-water layer in Pangnirtung Fiord may be a consequence of its shallow bathymetry compared with Kingnait Fiord. The deeper and wider sill in Kingnait Fiord may provide greater exchange of water with Cumberland Sound and support the retention of later life-stage copepodites. The opportunities for feeding appear to be greater in Kingnait Fiord with the presence of a shallow and deep prey layer. Furthermore, the quality of prey appears to be higher in Kingnait Fiord due to the greater abundance and biomass of Arctic taxa.

Similar to our net sampling, our analysis of stomach contents found that mostly *Calanus* spp. were consumed—representing two-thirds of the total number of zooplankton identified. Of the *Calanus* species, *C. hyperboreus* (30.0%) and *C. glacialis* (33.7%) were the most numerous. However, we were unable to identify down to the species level for 35.0% of all *Calanus* spp. due to missing urosome segments needed to stage individuals and identify species based on prosome size. Some of the unidentified species might be *C. finmarchicus*, which we found in the net samples. Other studies similarly found that the numerical abundance of bowhead whale stomach contents was dominated by copepods of the Calanidae family in the Beaufort Sea and Hudson Strait. However, they were unable to make species level identifications due to differences in preservation methods (freezing vs. formalin)^2^.

*Pseudocalanus* spp. was poorly represented in our stomach sample compared with the net samples. This may reflect diet selection of larger, more energy-rich organisms such as *Calanus* spp. whereby the whale was feeding on prey layers that contained proportionally low abundances of *Pseudocalanus* spp. or it may be an artefact of the small size of the organisms and the presumably quicker time required for digestion (e.g., effects of differential digestion^55^). As a consequence, it is possible that smaller species and earlier life-stages are underestimated from stomach content analysis and that these results represent the minimum number of species consumed. However, the species identifications from the stomach sample provide confirmation that the whales were consuming *Calanus glacialis* and *C. hyperboreus*, which is consistent with our interpretation of the vertical distribution of particles and foraging behaviour of the whales.

### Zooplankton vertical distribution

The presence of two discrete prey layers in Kingnait Fiord likely reflects the oceanographic conditions, zooplankton species diversity and life-history characteristics. The depth of maximum particle biomass in the surface waters occurred near the estimated mixed layer depth that was between 8 and 26 m (17 m on average based on n = 16 stations behind Kekertukdjuak Wesland) (Figs. 4 and 5). Under stratified conditions, phytoplankton concentrations should be greatest in warm, low-density, nutrient-rich surface waters near the mixed layer where light penetration is high^56,57^. As a result, actively feeding herbivorous copepods, such as early stage *Calanus finmarchicus*, would be expected to co-occur near the mixed layer where zooplankton feeding conditions are presumed greatest. Physical oceanographic features are also associated with North Atlantic right whale feeding activities as they routinely exploit diapausing life-stages of *Calanus finmarchicus* that are concentrated near the bottom mixed layer^58^. Consequently, obtaining simultaneous temperature and salinity data needed to estimate the mixed-layer depth is important for making inferences about the seasonal ontogeny of bowhead whale prey.

As the summer progresses and phytoplankton concentrations decrease due to grazing, later developmental stages of *Calanus finmarchicus* (e.g., CIV and CV) and early to late stages of multi-generational Arctic species such as *C. glacialis* and *C. hyperboreus* (e.g., CIII, CIV and CV) may begin their seasonal descent to near bottom depths for diapause^5,59–61^. Diapause is a form of dormancy, whereby copepods suppress their metabolic rates by occupying cooler water masses and reduce their swimming activity as a means to conserve their lipid reserves until the following spring when they ascend to surface waters to feed during the phytoplankton bloom^1,5,39,53,62^.

The deeper prey layer included particles that were comparatively larger (1.63 mm ECD) than those found in the near surface layer (1.21 mm ECD). We found that the spherical volume ($\frac{4}{3}\pi {r}^{3 } where\; r \; is \, \frac{ECD}{2})$ of zooplankton particles at depth (mean spherical volume was 2.27 mm^3^) were 2.44 times larger than those at the surface (0.93 mm^3^). These bigger particles were presumably the larger bodied Arctic copepods identified in the net tows. Particle measurements from an OPC are typically smaller than the actual copepod prosome measurements due to differences in the orientation and transparency of the organisms as they pass through the light beam^63^. For example, previous studies found that *Calanus finmarchicus* CV ECD was underestimated by ~ 30% using the OPC^64^. Given the likelihood of underestimation and the preponderance of early-life stage *C. glacialis* (e.g., CIV measures between 2.03–2.93 mm) and *C. hyberboreus* (e.g., CIII measures > 1.95 mm) (Table S7), it is feasible that the CIII and CIV organisms found in the net tows were represented by the comparatively larger particles detected by the OPC in the deep layer (190–225 m).

Predator avoidance and adaptation to seasonally predictable fluctuations in phytoplankton availability likely explain why Arctic copepods have either initiated diapause or undergone diel vertical migration. Diel vertical migration is a form of predator avoidance whereby small prey species such as zooplankton descend to depth (below the euphotoic zone) during daylight hours to avoid predation from visual predators^65–68^. It may be particularly beneficial for larger copepods that have accumulated considerable lipids to undergo diel vertical migration because the risk of predation outweighs the benefit of feeding during daylight hours^69^.

In the absence of day and night sampling, we can only speculate as to which mechanism was regulating copepod vertical movement. However, the Arctic species identified based on prosome lengths (Fig. S2 & Table S6) represent diapausing stages (e.g., CIV-CV for *C. glacialis* and CIII-CV for *C. hyperboreus*). Furthermore, previous studies in Disko Bay, Greenland found that both *C. glacialis* and *C. hyperboreus* terminated feeding even when phytoplankton remained available^57^, providing support that these organisms were engaged in diapause as opposed to diel vertical migration at the time of sampling. Diel patterns in bowhead whale diving behaviour were not observed in our study (S1), providing additional evidence that the sampled copepods at depth were undergoing diapause.

### Energetic trade-offs between prey layers

The variability in bowhead foraging dive depth may reflect a balancing of energetic trade-offs associated with diving and prey consumption^70,71^ which has been documented in rorqual species such as blue whales that employ divergent feeding strategies in response to vertical differences in prey density^72^. Bowheads may use shallow feeding dives on lower energy prey to complement the presumably reduced feeding times and potentially increased energy expenditure associated with deeper feeding on higher energy prey. Although the actual aerobic dive limit is unknown for balaenids (because logistical constraints prevent collecting blood samples to quantify post-dive lactate concentrations), the proportion of the total dive duration spent at the surface post-dive is considered to be an indicator of whether or not an animal has incurred a build-up of lactate in the blood due to anerobic metabolism that is being cleared during the post-dive surface interval^73,74^.

We examined the proportion of total dive duration spent at the surface post-dive for short-term, fine-scale tagged animals and found that it did not increase with increasing dive duration or depth. Consequently, it appears as though bowhead whales are within their calculated aerobic dive limit while conducting dives between 10 and 305 m that last between 0.65 and 28 min. Similarly, North Atlantic right whales (*Eubalaena glacialis*) were found to be within their aerobic dive limit when conducting foraging dives to ~ 120 m^45,58^. However, it is unknown how increased dive duration (> 28 min) may impact the aerobic dive limit of bowhead whales. It is reasonable to assume that bowheads may require additional time at the surface to physiologically recover after long periods of successive deep diving. Access to shallow aggregations of prey may provide animals with an energetic respite while feeding almost continuously^45^.

## Conclusions

The comparison of two adjacent fiords—one with bowhead whales and one without—revealed bowhead whale prey preferences and provided important insight about the foraging strategy in the Eastern Canadian Arctic. Most notably, it suggests that the whales preferred Kingnait Fiord over Pangnirtung Fiord because it had a deep prey layer with a higher biomass of Arctic taxa. Bowhead whales feeding in Kingnait Fiord may offset the presumably increased energy costs incurred from repeated deep dives by intermittently exploiting shallowly aggregated prey layers when they occur in high-abundances. Combining vertical prey sampling and multi-scale tagging thus revealed that bowhead whales exploit multi-depth prey layers and are flexible foragers. Together, these data provide the first confirmation that Cumberland Sound is a summertime foraging habitat for ECWG bowhead whales.

Our results further suggest that although prey quality and quantity are important to bowhead whale feeding, biomass is ultimately the most influential. The whales appeared to preferentially target deep aggregations of high biomass, which were likely comprised of a relatively low numerical abundance of higher energy Arctic taxa (e.g., *Calanus glacialis* and *C. hyperboreus*). However, in Disko Bay bowhead whales appeared to prefer higher numerical abundances of lower energy temperate/subarctic prey (e.g., *Calanus finmarchicus*) at depth^1^. Peak abundance of juvenile copepodites occurred in May/June for *Calanus hyperboreus,* July for *C. glacialis* and August for *C. finmarchicus*, suggesting that a change in the dominant species may impact the seasonal availability and biomass of prey^39^. Bowhead feeding depth in both habitats coincided with the depth of maximum biomass, suggesting that this is the best metric for determining where whales are likely to feed in the water column and for assessing habitat quality. Consequently, assessments of prey quality (e.g., energetic density of organisms) or quantity (e.g., numerical abundance of organisms) alone don’t appear to be the best predictors of bowhead whale foraging activity.

How bowhead whale foraging behaviour and energy balance may be affected by future changes in the species composition and abundance of their prey associated with climate change is unknown. There is a need to predict how future changes in environmental conditions are likely to alter the distribution and abundance of temperate/subarctic and Arctic copepods throughout the range of ECWG bowhead whales. One possible scenario may include a shift in species composition whereby *Calanus hyperboreus* and *C. glacialis* experience a movement poleward and *C. finmarchicus* dominates zooplankton assemblages in Cumberland Sound. However, it is unknown whether lower quality prey will be sufficient to support the energetic requirements of the population at its current numbers if temperate/subarctic species become dominant. Similarly, it is unknown whether bowheads will abandon their current habitat and move northward with the range shift in Arctic calanoid copepods as has occurred in the past^75^. Addressing these questions through modeling simulation will help us understand whether Cumberland Sound will continue to be an important summertime habitat for this segment of the population in the face of climate change.

## Methods

### Fine-scale bowhead whale dive data collection

Detailed foraging behaviour was studied using a modified version of the short-term dermal tag^76^ during August 2016. The dermal tag was equipped with a Lotek LAT1500 time-depth recorder (i.e., pressure sensor). A 36-kHz Vemco acoustic transmitter was used to assist in tracking the tagged whale during focal follows with a directional hydrophone. The dermal tag was adapted to include a Wildlife Computers SPOT6 satellite tag instead of a radio transmitter as previously used^76^ to facilitate tag retrieval for whales that were not focal followed after tagging. The tag was deployed using a compressed air launcher and attached using a 7.5 cm long needle (~ 0.6 cm in diameter) that was steam sterilized in an autoclave prior to use.

The tagging fieldwork for this study involved first observing the behaviour of individual animals and then selecting candidate whales for tagging purposes. Candidates were selected if they were not part of a mother-calf pair and were preferably 8 m or greater. Body length was visually estimated in meters using a small unmanned aerial system (UAS DJI Phantom Professional 3). Candidate whales also needed to exhibit behaviours indicative of foraging (e.g., high-fluking and long dives where bottom depth was ≥ 100 m) and this was assessed prior to tagging using boat-based and UAS observations. The position of the whale and flight altitude were automatically recorded while conducting focal follows with the UAS that was equipped with a global positioning system and altimeter. The average altitude of the UAS was 12.9 m (± 5.4 SD) and did not result in any observed change in whale behaviour despite the relatively low flight altitude. This suggests that the Phantom Pro can collect undisturbed behavioural data. The maximum distance flown away from the vessel was 1000 m (line-of-sight).^The UAS was hand-deployed and hand-retrieved from the vessel and flight times lasted between 8 and 12 min.^

Once a candidate whale was selected, the individual was approached by a 6-m aluminum vessel for tagging. For the majority of tagging events, the UAS was deployed to record video and images for photogrammetry and photo-identification. Close approaches to whales were conducted in a controlled manner at safe speeds to avoid disturbing the whale and to ensure that the approach could be terminated at any time.

Once the dermal tag was successfully deployed using the compressed air launcher, the tagged animal was photographed and/or videoed and some were subsequently focal followed while others were not. Photographs were taken of the tag site if possible and of the unique marks (e.g., scars, pigmentation, killer whale (*Orcinus orca*) rake marks^77^) on the whale’s body to avoid retagging the same animal. Focal followed animals were tracked using visual observations while at the surface (boat-based and UAS observations). While submerged, the bearing and real-time-depth of the tagged whale was monitored by detecting the acoustic pings of the Vemco transmitter using a directional hydrophone and acoustic receiver. The research vessel made every effort to stay far enough away (e.g., 500 m) from the tagged whale to avoid altering the animal’s behaviour but remained within range of the acoustic transmitter (1 km) at all times. While tracking the animal, GPS locations were continuously logged using a handheld global positioning system (GPS). These data were collected to reconstruct the spatial movements of the tagged animal and to coordinate prey sampling with the oceanographic sampling vessel. On several occasions, the tag placement was high enough on the whale’s back to permit satellite transmission and thus providing ARGOS locations during surfacings. The real-time dive depths of the tagged whale were recorded and relayed to the oceanographic sampling vessel. Vertical profiles with the OPC and CTD were conducted to the whale’s maximum dive depth using the oceanographic instrument package.

We focal-followed three animals for 0.83 to 9.23 h and found that the animals remained close to shore for the duration of the focal follows and displayed behaviours that were inconsistent with feeding, such as conducting shallow, non-fluking dives and occasionally engaging in rock-rubbing behaviour^78^. Given the lack of foraging behaviour during focal follows, we increased our vessel distance to the tagged whale from ~ 500 m to ~ 800 m and used the UAS to aerially follow the animal and record behaviour in real-time. The aerial footage failed to record any surface feeding activities, which was consistent with our boat-based observations. Occasionally, we observed individuals with slightly agape mouths in shallow rock-rubbing habitat. However, prior prey sampling in these areas showed extremely low zooplankton abundances suggesting that the whales were more likely engaged in thermoregulation activities than feeding^78^. Given the low density of vessels in Cumberland Sound and potentially low likelihood of habituation to human activities, we hypothesized that the behaviour of the focal followed animals was influenced by the presence of our vessel and subsequently excluded their dive data from our analysis. Consequently, a subset of animals were not focal followed and instead were immediately left upon tagging to reduce the post-tagging recovery time and mitigate any potential changes in behaviour caused by the presence of the focal follow vessel. For animals left after tagging, oceanographic data were collected in adjacent waters in Kingnait Fiord and thus the prey data were not as closely spatially co-located (e.g., not in the fluke print of a tagged animal but in adjacent waters several hundred meters or more away) with the tagged whale.

### Longer-term bowhead horizontal and vertical movement data collection

To record horizontal and vertical movements over longer spatial scales (days to weeks) we equipped 9 whales with long-term satellite telemetry tags outfitted with time-depth recorders (Wildlife Computers SPLASH MK10). The SPLASH tag provided information on date, time, location, and summary dive behaviour (e.g., depth, duration, shape). Wildlife Computers classified dive shape based on three broad categories such that V-shaped dives include those where ≤ 20% of the total dive duration was spent at maximum depth, U-shaped dives occurred where > 20% and ≤ 50% of the dive duration was spent at maximum depth and Square dives included those where > 50% of the dive duration was spent at maximum depth. Maximum dive depth (i.e., the bottom depth of a dive) is defined by Wildlife Computers as being a depth ≥ 80% of the maximum reading observed for each dive. The platform transmitter terminals (Ptts) were programmed to transmit up to 400 times a day every second hour during summer.

Three males and 6 females were tagged in Cumberland Sound between 20 and 28 August 2016. However, one tag failed to transmit during August (Ptt 148,499). Juvenile, sub-adult and adult animals were selected for tagging (excluding lactating females), which meant tagging animals ≥ 9 m long to avoid calves and those in mother-calf pairs. The SPLASH tag was attached using a  20 cm stainless steel anchor that penetrated the animals’ skin and blubber. The tag was deployed with an 8 m fiberglass pole, allowing for simultaneous collection of bowhead tissue from a 4 cm biopsy tip incorporated into the end of the pole; the sex of the tagged whale was determined from this biopsy. Prior to attachment, the anchor and biopsy tip were sterilized with a 1:10 bleach/water solution. The tags were deployed from a wooden canoe freighter and attached dorsally, behind the blow holes.

### Horizontal movement analysis

The Square Root Unscented Kalman Filter (SRUKF) was used to re-process the raw Argos data by Service Argos. This SRUKF algorithm uses a correlated random walk model to predict future positions based on an animals previous location and estimated error^79,80^. The Kalman Filter is preferable over the Least-Squares algorithm because it generally increases the number of positions while improving the accuracy of lower quality Argos locations (i.e., 0, A and B) that are common with large whale studies^79,80^. We then ran the SRUKF filtered data through a speed filter using the vmask function in the argosfilter package in R^81^. This function identifies improbable positions based on a maximum swimming speed. Argos locations that resulted from an animal swimming above > 2 m s^−1^ were removed from our analysis.

To estimate individual animal movement, determine the behavioural state associated with the Argos position (i.e., probable foraging or transiting) and quantify location error, we fit a HSSSM^82,83^ using the bsam package in R^81^. We fitted a correlated random walk model (CRW) that switched between two behavioural states that reflected area restricted movements (ARM) and traveling^82^, whereby the associated behavioural states differ in mean turn angle and swimming speed^82^. For example, ARM (which is thought to reflect predators searching for and consuming prey^84–88^) reflected low swimming speeds and high turning angles, while traveling consisted of faster, more linear movements (such as those associated with seasonal migrations). We fit the HSSSM to each data set containing individual specific location data with 40,000 Monte Carlo Markov Chain (MCMC) iterations. We dropped the first 30,000 (i.e., burn-in) and retained every 10th sample from the remaining 10,000, resulting in a total of 1000 samples per chain (n = 2 chains).

Bowhead whale behavioural states (*b*) associated with HSSSM predicted locations were classified based on mean estimates from the MCMC samples, whereby *b* = 1 was assumed to represent transiting behaviour and *b* = 2 reflected ARM. We used the same thresholds as previous studies^79,89^ such that predicted locations with mean estimates of b > 1.75 were assumed to indicate ARM; *b* < 1.25 reflected transient behaviour; values between *b* ≥ 1.25 and *b* ≤ 1.75 were unclassified. To exclude inaccurate location data from our analysis, we filtered the predicted locations from the HSSSM by removing locations that resulted from gaps exceeding 4 consecutive days based on the raw SRUKF data.

### Vertical movement analysis

To determine where the SPLASH tagged whales were feeding in the water column, we analyzed the summary time-depth-recorder data. For each dive, we obtained measurements of dive duration, shape (V, U, Square or unknown) as well as minimum and maximum dive depth. For comparison with the fine-scale TDR data, we defined dives as vertical excursions to ≥ 10 m. Behaviour may be inferred based on dive shape whereby previous studies for North Atlantic right whales and bowhead whales found that V-shaped dives reflected search behaviour while Square and U-shaped dives where animals increased the time they spent at maximum depth reflected feeding dives^1,5,21,58^.

We filtered the bowhead whale dive behaviour data based on the predicted HSSSM location data to differentiate dives that occurred inside Cumberland Sound with those that occurred inside Kingnait Fiord. We then merged the vertical dive data with the HSSSM location data by matching dates between the two data sets (horizontal and vertical). We assumed that animals in Kingnait Fiord did not make daily excursions to Cumberland Sound and vice versa. Consequently, all dives were assumed to occur within the same habitat where HSSSM predicted locations occurred. To compare bowhead whale dive behaviour with the vertical distribution of zooplankton, we excluded bowhead whale dives that occurred after dusk and before dawn because all zooplankton sampling was conducted during daylight hours. We filtered the dive data using the time of sunrise and sunset for Iqaluit, Nunavut based on the median sampling dates of August 26 and September 15, 2016.

We investigated whether bowhead whale dive depth and duration were impacted by dive shape, month and time of day (day or night) using linear-mixed effects models with the lme statistical function in R 3.6.1 (R Development Core Team 2020). To test for differences in bowhead whale dive behaviour based on dive shape and month, we used dive shape and month as fixed-effects and maximum depth (m) and dive duration (mins) for all dives occurring during August and September 2016 as the response variables. We also tested for diel patterns in bowhead dive behaviour by separating dives that occurred during the day and night in late August 2016 and used dive shape and day/night as a fixed-effect and maximum depth (m) as the response variable. Using a step-wise modeling approach, we fit several nested linear mixed-effects models and compared each model using likelihood ratio tests to examine how dive shape, month and time of day affect bowhead whale dive depth and duration. We also used the Akaike’s information criterion (AIC) to indicate model support and selected the model with the lowest AIC value as being the ‘best’ model. We included a hierarchical error structure of individual (Ptt), month, and day, along with a continuous autoregressive process within day because our data were irregularly spaced in time (i.e., CAR(1) process^90^). When comparing the null models with and without the random effects structure we found support for the more complex error structure (i.e., lower AIC and supported by Likelihood Ratio Tests). We confirmed this error structure as well as normality using plots of standardized residuals.

### Optical plankton counter particle size, abundance and biomass

We used a Focal Technologies OPC-1T optical plankton counter (OPC) to obtain a vertical profile of zooplankton abundance by particle size^64^. We mounted the instrument in the center of an aluminum protective sampling cage and lowered it vertically through the water column at 0.75 m s^−1^ to approximately 10 m above the sea bottom. The OPC has a lower ECD size detection limit of 0.25 mm which should accurately detect most species and life-stages of copepods^64^. The OPC was used to determine the depth where maximum zooplankton abundances occurred. We analyzed the OPC downcast data only, in 4 m depth bins to calculate descriptive statistics of: (1) equivalent circular diameter (mm); and (2) particle abundance (particles m^−3^). We further excluded all OPC measurements less than 1.0 mm ECD so as to remove detritus and small copepods (e.g., *Pseudocalanus* spp.). We used Ocean Data View software to visualize our OPC data and Data-Interpolating Variational Analysis (DIVA) was selected for spatial interpolation of the in situ OPC data to generate contour plots of particle abundance and mean particle size.

The physical oceanographic characteristics of Kingnait Fiord were determined by collecting co-located physical oceanographic data with each OPC deployment using a Seabird SBE19Plus conductivity, temperature, and depth (CTD) profiler. CTD data were processed using Seabird software, and temperature and salinity plots were generated using the downcast data for each profile. For visualization purposes, we aggregated the temperature and salinity data into 1 m depth bins. To estimate the base depth of the mixed layer—the layer of uniform density near the surface where phytoplankton and feeding zooplankton may concentrate^91–93^, we derived the potential density of the mixed layer using salinity (PSU) and temperature (°C) measurements from the CTD profiles using Ocean Data View. We then used the threshold difference method to determine the depth where the potential density changed by ~ 0.01 kg m^−3^ (using a range of ≥ 0.01 and ≤ 0.02 kg m^−3^) relative to the ocean surface density.

To gain an understanding of how zooplankton biomass varied throughout the water column, we used the OPC particle measurements and counts to calculate biomass concentration (mg m^−3^) by: (1) converting ECD (mm) into wet weight (mg) using:

$${W}_{wet}=\frac{4}{3}\pi {\left(\frac{ECD}{2}\right)}^{3}\rho$$

where ${W}_{wet}$ is the wet weight of a particle (mg) and ρ is the particle density (mg m^−3^)^94^; (2) summing ${W}_{wet}$ for each particle with ECD ≥ 1.0 mm within a specified depth range; and (3) dividing this total weight (mg) by the volume of water sampled (m^-3^). We assumed for this calculation that a sphere accurately represents particle volume and that each particle has a density of 1 mg mm^−3^^94^.

### Zooplankton collection net sampling

We determined species composition from net collected zooplankton samples taken during August 2016 in two fiords in Cumberland Sound, Nunavut—one where bowhead whales are seldom seen (Pangnirtung Fiord; 66° 09′ 22.0″ N and 65° 43′ 25.3″ W) and another where bowheads are routinely observed (Kingnait Fiord; 65° 57′ 07.1″ N and 65° 19′ 46.5″ W) (Table S1). Zooplankton samples were collected throughout the water column for enumeration and species identification using a standard conical 333 µm mesh net (60 cm in diameter). Oblique samples were collected by towing the weighted net up to the surface at an oblique angle from various depths, and vertical samples were obtained using the same net by lowering it to ~ 5 m above the sea floor and then hauling it straight back to the surface using an auto-hauler. During the oblique and vertical sampling, the net was hauled at ~ 1 m s^−1^. The mouth of the net was instrumented with a Vemco acoustic transmitter (V16Ps 51 kHz or 84 kHz) capable of sampling down to 340 m and 680 m respectively. These transmitters were used with an omni-directional hydrophone and Vemco acoustic receiver to determine the net sampling depth in real-time.

Once the zooplankton nets were brought onboard the ship, the volume of filtered water was recorded using a flow meter and all nets were sprayed down with seawater. Sampled organisms were concentrated into the attached cod end bucket and subsequently filtered through a 333 μm mesh sieve and then transferred to a 250 mL sample jar. These samples were fixed immediately after collection and preserved in a 5% buffered formaldehyde-seawater solution.

Zooplankton species identification and enumeration was conducted in the laboratory. Each net-collected sample was filtered through a 333 μm mesh sieve, rinsed and transferred to a beaker, and diluted with water. The sample volume was recorded and a homogenous aliquot (i.e., sub-sample of known volume) was obtained using a Hensen-stemple pipette. For dense samples, a Folsom plankton splitter was used for sub-sampling purposes. The total number of times each sample was split (0–8 times) was dependent upon the total number of sample organisms. Each aliquot contained a minimum of 200 calanoid copepods and each organism was identified to the lowest possible taxon (e.g., species and genus for calanoid copepods) and life-stage for *Calanus* spp. and *Pseudocalanus* spp. using a dissecting microscope.

Due to the morphological similarity between *Calanus hyperboreus*, *C. glacialis* and *C. finmarchicus*^95,96^*,* first, the life stage of each organism was determined based on the number of urusomal segments and the swimming legs, and then prosome lengths were measured^54^ for all *Calanus* spp. using a dissecting microscope, stage micrometer and ocular micrometer. All organisms were measured from the same orientation (right lateral side down) to reduce measurement variability. *Calanus* species were assigned based on previously reported species-specific prosome size ranges^39^. However, there is likely some overlap in prosome length between species^54^, which introduces error into the identification of early life-stages. Prosome measurements were not made for *Pseudocalanus* spp. and instead mean prosome lengths for early and late staged organisms were determined from values reported in the literature.

Zooplankton biomass was calculated as weight (mg C) for *Calanus* spp. and *Pseudocalanus* spp. using a previously established allometric length–weight relationship:

$${W}_{dry}=a{L}^{b}$$

where ${W}_{dry}$ is weight (mg C), $L$ is prosome length (μm) and a = 0.0048 and b = 3.5687 for *Calanus finmarchicus* and *C. glacialis*^39^*,* a = 0.0014 and b = 3.3899 for *C. hyperboreus*^97^ and a = 6.12E-11and b = 2.7302 for *Pseudocalanus* spp.^98^. We used our measured prosome lengths for all life-stages of *Calanus* spp. to calculate biomass. To estimate *Pseudocalanus* spp. biomass, we used average prosome lengths for early (CI-CIV: 596.8 μm) and late (C5-Adult; 1009.9 μm)^37^ life-stages from the literature and the number of enumerated *Pseudocalanus* spp. copepods per station in our samples.

### Bowhead stomach contents

Bowhead whale diet composition was directly determined from the stomach contents of a sub-adult female bowhead whale harvested in Kingnait Fiord (Cumberland Sound, Nunavut) on 14 September 2016. In collaboration with local hunters, we obtained a 500 mL sub-sample of the stomach contents to qualitatively identify prey species. Due to the size of the stomach, potentially large volume of prey and logistical challenges associated with collecting stomach contents from large whales, we were not able to quantitatively determine the volume of consumed prey. The 500 mL prey sample was preserved in 5% buffered formaldehyde for subsequent species identification using a dissecting microscope. The same methods employed for net collected samples were used for species identification of stomach collected organisms.

### Research guidelines

We applied previously approved standard operating procedures and guidelines for bowhead whale tagging and biopsying created by Fisheries and Oceans Canada and Woods Hole Oceanographic Institution. We received approval for all research relating to bowhead whales including tagging, biopsying, aerial drone photography and boat-based observations from animal welfare and ethics boards at the University of British Columbia and Fisheries and Oceans Canada. The unmanned aerial systems data were collected under Special Flight Operation Certificate File Number 5812-11-682, ATS 16-17-00014027, RDIMS 12044419 and approved by the University of British Columbia Animal Care Committee (Animal Care Amendment A14-0064-A002). Bowhead whale behavioural data were collected under Department of Fisheries and Oceans License to Fish for Scientific Purposes S-12/13-1014-NU, S-13/14-1009-NU and S-16/17 1005-NU and Animal Use Protocol FWI-ACC-2016-09.

## Supplementary information

Supplementary Information.

## Data Availability

All data that are not contained within the supplementary information tables, will be made available through an online data repository.
