# Supplementary material for: Bowhead whales use two foraging strategies in response to fine-scale differences in zooplankton vertical distribution
Source: Sci Rep. 2020 Nov 20;10:20249. doi: 10.1038/s41598-020-76071-9 (PMC7680138; doi:10.1038/s41598-020-76071-9)
Supplement: Supplementary file 1 — Supplementary Information. [file 41598_2020_76071_MOESM1_ESM.docx]

**Bowhead whales use two foraging strategies in response to fine-scale differences in zooplankton vertical distribution**

Sarah M. E. Fortune^1,3^,* Steven H. Ferguson^2^, Andrew W. Trites^1^, Justine M. Hudson^2^ and Mark F. Baumgartner^3^.

*^1^Department of Zoology and Marine Mammal Research Unit, Institute for the Oceans and Fisheries, University of British Columbia, Vancouver, British Columbia V6T 1Z4, Canada*

*^2^Fisheries and Oceans Canada, Freshwater Institute, Winnipeg, Manitoba R3T 2N2, Canada*

*^3^Biology Department, Woods Hole Oceanographic Institution, Woods Hole, Massachusetts 02543-1050, United States of America*

[s.fortune@oceans.ubc.ca*](mailto:s.fortune@oceans.ubc.ca*)

**Supplementary Information**


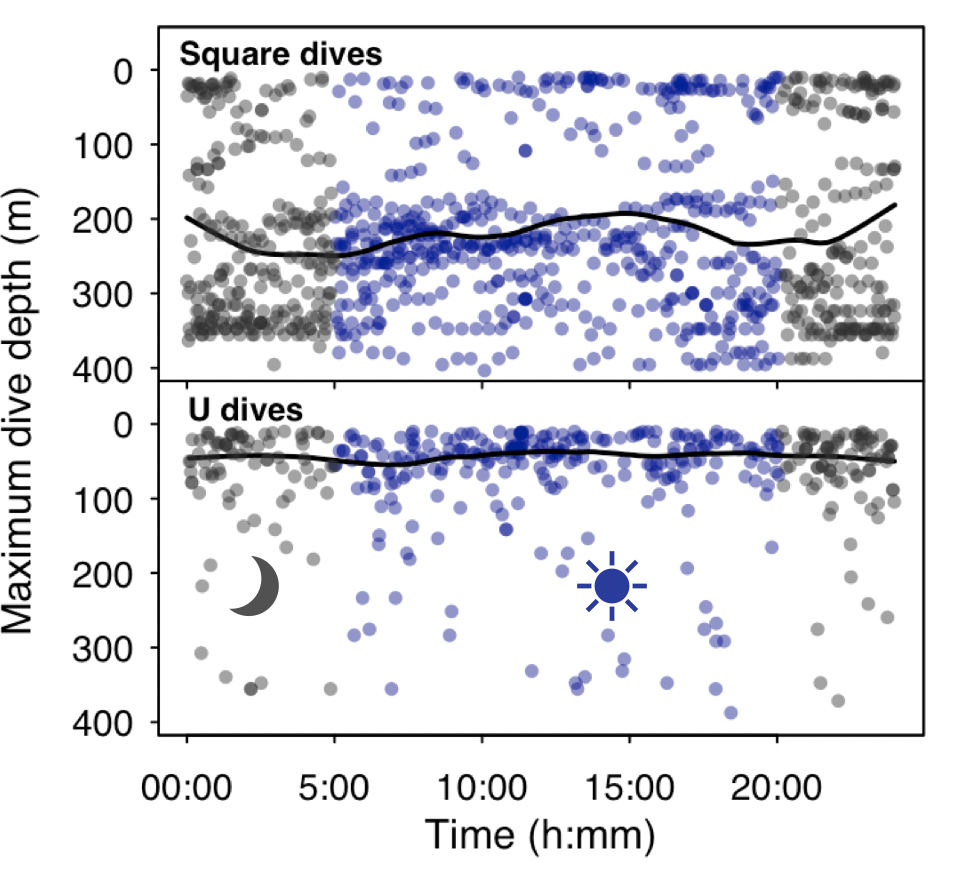


Figure S1. Maximum dive depth (m) of Square (top) (n = 537 day and 439 night) and U (bottom) (n = 255 day and 167 night) shaped dives during daylight (•) and darkness (•) for 8 bowhead whales during August in Cumberland Sound 2016. A lowess curve was fit to the Square and U-shaped dive data for trend visualization purposes.

**
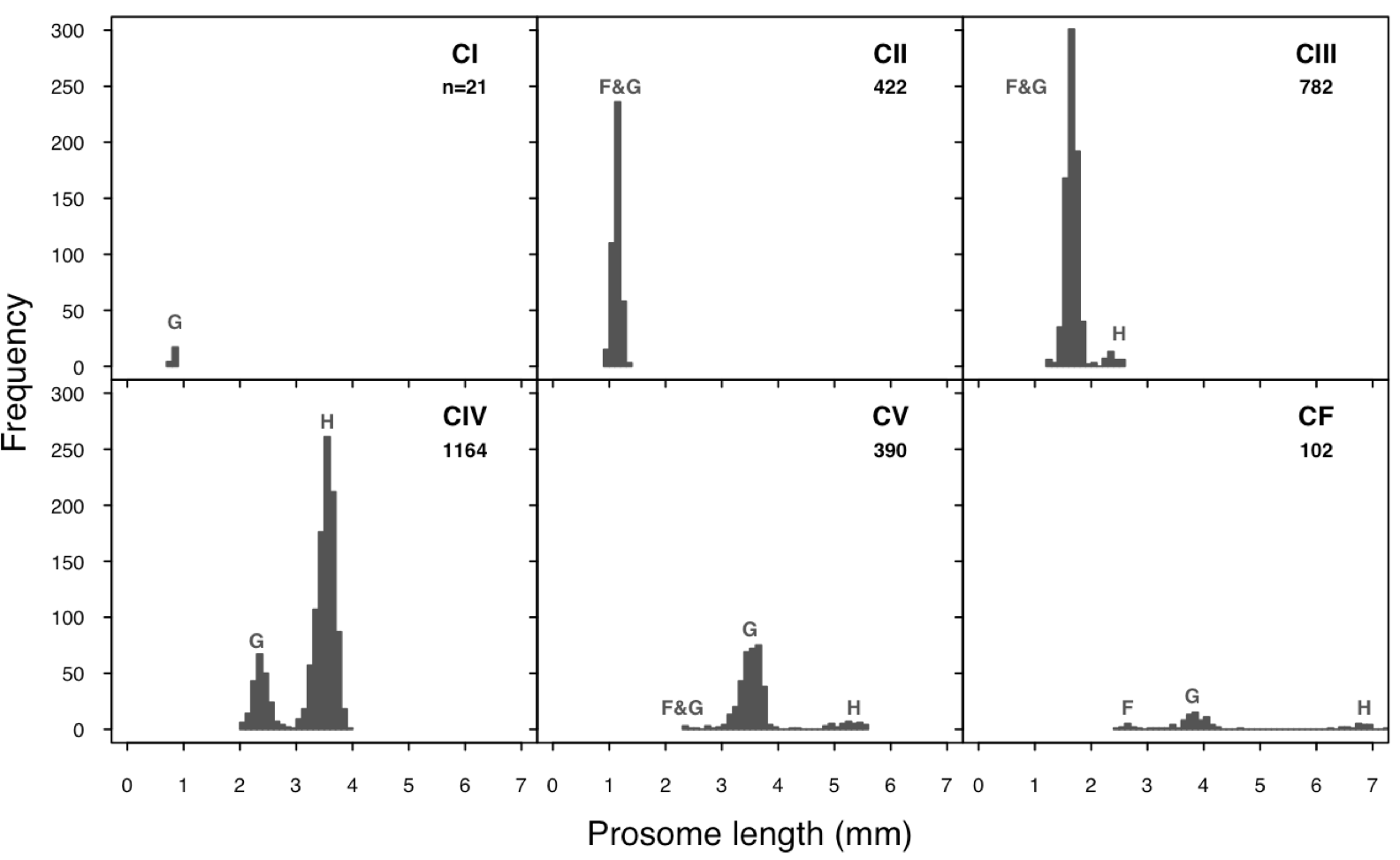
**

Figure S2: Distribution of prosome lengths by life-stages for all 3 *Calanus* spp. identified and enumerated from 20 net samples collected in Kingnait Fiord.

These measurements differentiated the three morphologically similar *Calanus* spp. based on the species-specific size ranges determined by Madsen *et al*. (2001) for *Calanus finmarchicus* (F), *C. glacialis* (G) and *C. hyperboreus* (H). Some overlap in size ranges is likely to occur between species, making identifications particularly challenging for early-stages such as CII and CIII *C. finmarchicus* and *C. glacialis*.

Table S1: Summary information for the 6 bowhead whales that were tagged with the fine-scale TDR in Kingnait Fiord during August 2016.

Three of these animals were left upon tagging (TDRs 2, 5 and 6) and their dive data was subsequently analyzed to determine where they were likely feeding in the water column; focal follows were conducted on the other three whales. The date is when the animal was tagged in Kingnait Fiord and the hours indicates the amount of time of behavioural data collection.

| **TDR** | **Date** | **Time (GMT)** | **Hours** | **Longitude** | **Latitude** |
| --- | --- | --- | --- | --- | --- |
| 1 | 2016-08-15 | 17:28 | 0.827 | -65.614 | 66.192 |
| 2 | 2016-08-26 | 16:29 | 15.62 | -65.500 | 66.124 |
| 3 | 2016-08-27 | 18:08 | 3.196 | -65.603 | 66.074 |
| 4 | 2016-08-28 | 16:02 | 9.233 | -65.492 | 66.147 |
| 5 | 2016-08-29 | 17:20 | 12.654 | -65.210 | 66.181 |
| 6 | 2016-08-29 | 19:58 | 8.5197 | -65.375 | 66.181 |

Table S2: Summary data for bowhead whale equipped with longer-term and coarser scale satellite telemetry time-depth recorder tags (SPLASH tags) during August 2016. Five animals resided exclusively in Kingnait Fiord (Ptts 148499, 148502, 148504, 148505, 126499).

The date is when the individual was tagged and Aug and Sept (days) is the number of days of telemetry and dive data during each month. Body length (m) was estimated based on visual observation of animals from the vessel (e.g., tip of snout to fluke notch). Sex was determined for these animals using genetic analysis of the biopsy samples obtained with each tag deployment. The longitude and latitude are provided for the location where the animal was tagged.

| **Ptt** | **Date** | **Time**  **(GMT)** | **Aug (days)** | **Sept**  **(days)** | **Longitude** | **Latitude** | **Length (m)** | **Sex** |
| --- | --- | --- | --- | --- | --- | --- | --- | --- |
| **148499** | 2016-08-20 | 16:55 | 0 | 3 | -65.487 | 65.771 | 9 | M |
| 148500 | 2016-08-20 | 17:19 | 11 | 13 | -65.497 | 65.742 | 9 | F |
| **148502** | 2016-08-23 | 21:12 | 8 | 30 | -65.299 | 65.921 | 9 | M |
| 148503 | 2016-08-23 | 22:41 | 4 | 22 | -65.316 | 65.944 | 10 | F |
| **148504** | 2016-08-23 | 23:06 | 8 | 19 | -65.275 | 65.926 | 8.5-9 | F |
| **148505** | 2016-08-26 | 18:14 | 2 | 15 | -65.260 | 65.930 | 10 | M |
| 148506 | 2016-08-27 | 15:28 | 4 | 14 | -65.265 | 65.929 | 10 | F |
| **126499** | 2016-08-28 | 14:45 | 3 | 30 | -65.286 | 65.923 | 11 | F |
| 126500 | 2016-08-28 | 14:45 | 2 | 30 | -65.286 | 65.923 | 10 | F |

Table S3: The total number of dives (n dives) and SPLASH tagged animals (n animals) during daylight hours (between sunrise and sunset) by shape and month in Cumberland Sound and Kingnait Fiord.

During August two animals (Ptt 126500 and 148506) conducted only a single V-shaped dive in Cumberland Sound (CS). Similarly, Ptt 148505 conducted only one U and V-shaped dive during September in Kingnait Fiord (KF). Depth and duration reflect the weighted mean maximum dive depth (m) and associated minimum dive duration (mins).

| **Month** | **Location** | **Shape** | **Depth (m)** | **Duration (mins)** | **n dives** | **n animals** |
| --- | --- | --- | --- | --- | --- | --- |
| August | CS | Square | 214.52± 28.74 | 18.36 ± 1.52 | 537 | 8 |
| August | CS | U | 72.41 ± 29.03 | 9.01 ± 2.20 | 255 | 7 |
| August | CS | V | 69.20 ± 42.40 | 11.10 ± 3.00 | 41 | 7 |
| September | CS | Square | 215.54 ± 38.77 | 21.29 ± 2.61 | 1910 | 9 |
| September | CS | U | 84.37 ± 18.66 | 10.13 ± 1.68 | 322 | 8 |
| September | CS | V | 120.16 ± 60.85 | 14.13 ± 3.60 | 65 | 8 |
| August | KF | Square | 207.73 ± 13.44 | 18.08 ± 1.66 | 241 | 4 |
| August | KF | U | 76.17 ± 25.32 | 9.65 ± 1.63 | 176 | 3 |
| August | KF | V | 76.67 ± 38.81 | 11.66 ± 2.74 | 29 | 3 |
| September | KF | Square | 240.01 ± 19.69 | 22.89 ± 1.94 | 1125 | 5 |
| September | KF | U | 94.68 ± 18.76 | 10.48 ± 1.68 | 172 | 4 |
| September | KF | V | 169.46 ± 58.94 | 16.69 ± 2.87 | 28 | 4 |

Table S4: Linear mixed-effects models to determine the impacts of dive shape (Square, U, V and unknown), month (August and September) and time of day (day or night) on dive duration and maximum depth. The null model includes no fixed effects. The random effects are consistent across models and included Ptt, month and day in a hierarchical order for models 1-5 and Ptt and day only for models 6-7. Model selection was based on the likelihood ratio tests (LRT), the AIC score (AIC) and

change in AIC (Δ AIC) and these metrics are relative to the model earlier in the list for dive duration and for maximum depth following stepwise selection. Instances where there are no fixed-effects, LR test or Δ AIC values are noted (~).

| **Model** | **Fixed** | **df** | **AIC** | **LR test** | **Δ AIC** |  |
| --- | --- | --- | --- | --- | --- | --- |
| **August and September** | |  |  |  |  |  |
| **Dive duration (mins)** | |  |  |  |  |  |
| null | ~ | 5708 | 86324.5 | ~ | ~ |  |
| 1 | Shape | 5706 | 85110.3 | 1218.22 (p < .0001) | 1214.2 |  |
| 2 | Shape+Month | 5706, 7 | 85107.5 | 4.791 (p = 0.0286) | 2.8 |  |
| 3 | Shape*Month | 19967,31 | 301258.6 | 1217.196 (p < .0001) | 1199.2 |  |
| **August and September** | |  |  |  |  |  |
| **Maximum dive depth (m)** | |  |  |  |  |  |
| null | ~ | 5708 | 68922.7 | ~ | ~ |  |
| 4 | Shape | 5706 | 68504.3 | 422.5 (p < .0001) | 418.4 |  |
| 5 | Shape+Month | 5706, 7 | 68506.1 | 0.169 (p = 0.6809) | -1.8 |  |
| **August only** |  |  |  |  |  |  |
| **Maximum dive depth (m)** | |  |  |  |  |  |
| null | ~ | 1380 | 17067.2 | ~ | ~ |  |
| 6 | Shape | 1379,7 | 16860.7 | 210.45 (p < .0001) | 206.5 |  |
| 7 | Shape+day.night | 1379,8 | 16862.1 | 0.396 (p = 0.5293) | -1.4 |  |

Table S5: Summary of all quantitative net samples collected using a 333 μm mesh net.

Samples were obtained in the presence and absence of bowhead whales and some were collected directly in the fluke print (i.e., location where an animal conducted a fluking dive). Two hauling methods (vertical and oblique) were used to obtain prey samples as indicated by the tow type. The longitudes and latitudes are provided (decimal degrees) for the locations where the net sample was collected by area (Pangnirtung Fiord and Kingnait Fiord).

| **Date** | **Whales** | **Fluke print** | **Tow type** | **Longitude** | **Latitude** | **Area** |
| --- | --- | --- | --- | --- | --- | --- |
| 2016-08-05 | no | no | vertical | -65.558 | 66.254 | Pangnirtung Fiord |
| 2016-08-05 | no | no | vertical | -65.556 | 66.256 | Pangnirtung Fiord |
| 2016-08-05 | no | no | vertical | -65.555 | 66.257 | Pangnirtung Fiord |
| 2016-08-05 | no | no | vertical | -65.552 | 66.266 | Pangnirtung Fiord |
| 2016-08-07 | no | no | vertical | -65.942 | 66.067 | Pangnirtung Fiord |
| 2016-08-10 | no | no | vertical | -66.031 | 66.102 | Pangnirtung Fiord |
| 2016-08-10 | yes | yes | vertical | -65.393 | 65.925 | Kingnait Fiord |
| 2016-08-15 | no | no | vertical | -65.553 | 65.930 | Kingnait Fiord |
| 2016-08-15 | no | no | oblique | -65.331 | 65.961 | Kingnait Fiord |
| 2016-08-16 | yes | yes | vertical | -65.448 | 65.929 | Kingnait Fiord |
| 2016-08-16 | no | no | oblique | -65.277 | 65.933 | Kingnait Fiord |
| 2016-08-16 | no | no | oblique | -65.283 | 65.935 | Kingnait Fiord |
| 2016-08-16 | no | no | oblique | -65.281 | 65.937 | Kingnait Fiord |
| 2016-08-19 | yes | no | vertical | -65.467 | 65.917 | Kingnait Fiord |
| 2016-08-19 | yes | no | vertical | -65.315 | 65.968 | Kingnait Fiord |
| 2016-08-20 | no | no | vertical | -65.291 | 65.932 | Kingnait Fiord |
| 2016-08-19 | yes | no | vertical | -65.394 | 65.951 | Kingnait Fiord |
| 2016-08-23 | no | no | vertical | -65.505 | 65.923 | Kingnait Fiord |
| 2016-08-23 | no | no | vertical | -65.414 | 65.892 | Kingnait Fiord |
| 2016-08-23 | no | no | vertical | -65.360 | 65.936 | Kingnait Fiord |
| 2016-08-23 | no | no | vertical | -65.449 | 65.862 | Kingnait Fiord |
| 2016-08-23 | no | no | vertical | -65.467 | 65.867 | Kingnait Fiord |
| 2016-08-26 | no | no | vertical | -65.475 | 65.818 | Kingnait Fiord |
| 2016-08-27 | no | no | vertical | -65.415 | 65.914 | Kingnait Fiord |
| 2016-08-27 | no | no | vertical | -65.438 | 65.887 | Kingnait Fiord |
| 2016-08-28 | no | no | vertical | -65.524 | 65.943 | Kingnait Fiord |

Table S6: Proportion (Mean ± SD) of total abundance (orgs m^-3^) of the four most common zooplankton species (*Calanus hyperboreus*, *C. glacialis*, *C. finmarchicus* and *Pseudocalanus* spp.) by life stage for net samples collected in Kingnait Fiord.

Early life stage includes copepodites stage I to IV and late represents life-stages between V and adult (male and female).

| **Species** | **Life stage** | **Proportion** |
| --- | --- | --- |
| *Calanus hyperboreus* | Early | 17.13± 7.04 |
| *Calanus hyperboreus* | Late | 1.11 ± 1.20 |
| *Calanus glacialis* | Early | 18.74 ± 11.60 |
| *Calanus glacialis* | Late | 7.40 ± 5.39 |
| *Calanus finmarchicus* | Early | 7.10± 4.90 |
| *Calanus finmarchicus* | Late | 0.28 ± 0.40 |
| *Pseudocalanus* spp. | Early | 5.02 ± 2.46 |
| *Pseudocalanus* spp. | Late | 43.21 ± 10.11 |

Table S7: Percent of total abundance (orgs m^-3^) and biomass (mg C m^-3^) for the most common species from net collected samples in Pangnirtung Fiord (n = 6) and Kingnait Fiord (n = 20). Mean values and standard deviations are reported.

| **Species** | **Location** | **% Abundance** | **% Biomass** |
| --- | --- | --- | --- |
| *Pseudocalanus spp.* | Pangnirtung Fiord | 52.9 ± 11.15 | 23.4 ± 10.6 |
| *Calanus hyperboreus* | Pangnirtung Fiord | 5.5 ± 5.15 | 12.3 ± 9.65 |
| *Calanus glacialis* | Pangnirtung Fiord | 17.7 ± 8.13 | 39.9 ± 9.61 |
| *Calanus finmarchicus* | Pangnirtung Fiord | 23.9 ± 9.16 | 27.1 ± 15.6 |
| *Pseudocalanus spp.* | Kingnait Fiord | 48.2 ± 10.21 | 6.90 ± 2.93 |
| *Calanus hyperboreus* | Kingnait Fiord | 18.2 ± 7.02 | 20.8 ± 9.3 |
| *Calanus glacialis* | Kingnait Fiord | 26.1 ± 8.13 | 68.6 ± 10.8 |
| *Calanus finmarchicus* | Kingnait Fiord | 7.4 ± 4.79 | 3.7 ± 3.9 |

Table S8: Summary zooplankton abundances (orgs per m^-3^) and dry weights (mg C m^-3^) calculated first by early (CI-CIV) and late life-stage (CV-Adult) per species and then combined for each species (*Calanus hyperboreus*, *C. glacialis, C. finmarchicus* and *Pseudocalaunus* spp.).

Samples shaded in grey are from Pangnirtung Fiord and the unshaded samples were collected in Kingnait Fiord The fished volume is the total water filtered during the *oblique* or vertical net tows. Total abundance is the number of all staged *Calanus* spp. and *Pseudocalanus* spp. per cubic meter and the total dry weight is the total biomass (mg C m^-3^) of these organisms. Data were separated between sampling locations—Pangnirtung Fiord and Kingnait Fiord and mean and standard deviations (SD) for each measurement is present per area. Note that zooplankton abundance (orgs m^-3^) and dry weight (mg C m^-3^) reflect integrated water column net tows.

| **Date** | **Fished volume (m^-3^)** | **Total abund (orgs m^-3)^** | **Total**  **dry weight**  **(mg C m^-3^)** | ***C. hyp* abund (orgs m^-3^)** | ***C. hyp***  **dry weight (mg C m^-3^)** | ***C. glac* abund (orgs m^-3^)** | ***C. glac***  **dry weight**  **(mg C m^-3^)** | ***C. fin* abund**  **(orgs m^-3^)** | ***C. fin***  **dry weight**  **(mg C m^-3^)** | ***Pseudo* abund**  **(orgs m^-3^)** | ***Pseudo***  **dry weight (mg C m^-3^)** |
| --- | --- | --- | --- | --- | --- | --- | --- | --- | --- | --- | --- |
| 16-08-05 | 14.13 | 71.34 | 1.17 | 3.82 | 0.18 | 8.92 | 0.36 | 8.49 | 0.17 | 53.93 | 0.47 |
| 16-08-05 | 9.82 | 106.63 | 1.51 | 1.22 | 0.06 | 11.61 | 0.47 | 36.36 | 0.72 | 57.44 | 0.46 |
| 16-08-05 | 9.81 | 80.71 | 1.29 | 1.22 | 0.06 | 9.17 | 0.37 | 26.60 | 0.53 | 43.72 | 0.33 |
| 16-08-05 | 10.40 | 91.43 | 1.53 | 1.44 | 0.07 | 14.71 | 0.60 | 25.38 | 0.50 | 49.90 | 0.36 |
| 16-08-07 | 12.56 | 75.48 | 1.98 | 9.55 | 0.44 | 21.50 | 1.01 | 14.81 | 0.29 | 29.62 | 0.24 |
| 16-08-10 | 21.48 | 43.77 | 1.35 | 4.84 | 0.33 | 11.92 | 0.69 | 7.64 | 0.15 | 19.37 | 0.17 |
| **Mean** | **13.03** | **78.86** | **1.47** | **3.68** | **0.19** | **12.97** | **0.58** | **19.88** | **0.40** | **42.33** | **0.34** |
| **SD** | **4.48** | **20.93** | **0.28** | **3.25** | **0.16** | **4.68** | **0.24** | **11.42** | **0.23** | **14.91** | **0.12** |
| 16-08-10 | 60.19 | 26.63 | 2.34 | 5.12 | 0.37 | 3.84 | 1.80 | 0.35 | 0.01 | 17.33 | 0.16 |
| 16-08-15 | 70.93 | 17.17 | 1.57 | 2.11 | 0.25 | 5.50 | 1.23 | 1.10 | 0.02 | 8.46 | 0.07 |
| *16-08-15* | *94.95* | *15.50* | *2.04* | *3.12* | *0.54* | *2.44* | *1.39* | *0.42* | *0.02* | *9.52* | *0.09* |
| 16-08-16 | 56.52 | 19.75 | 1.45 | 4.67 | 0.40 | 2.87 | 0.90 | 1.49 | 0.05 | 10.72 | 0.09 |
| *16-08-16* | *28.18* | *28.11* | *0.80* | *6.81* | *0.31* | *7.52* | *0.31* | *4.97* | *0.10* | *8.80* | *0.08* |
| *16-08-16* | *27.58* | *53.08* | *2.44* | *14.36* | *0.75* | *14.50* | *1.41* | *5.66* | *0.11* | *18.56* | *0.17* |
| *16-08-16* | *28.26* | *21.16* | *1.12* | *6.65* | *0.31* | *5.38* | *0.70* | *2.55* | *0.05* | *6.58* | *0.06* |
| 16-08-19 | 54.54 | 27.72 | 0.63 | 0.00 | 0.00 | 10.67 | 0.43 | 5.06 | 0.10 | 11.99 | 0.10 |
| 16-08-19 | 95.52 | 21.03 | 2.14 | 3.74 | 0.35 | 3.08 | 1.66 | 0.22 | 0.00 | 14.00 | 0.13 |
| 16-08-20 | 29.96 | 35.25 | 1.85 | 2.60 | 0.12 | 15.22 | 1.53 | 4.41 | 0.09 | 13.02 | 0.12 |
| 16-08-19 | 89.84 | 25.38 | 3.90 | 4.94 | 0.46 | 6.81 | 3.30 | 0.40 | 0.01 | 13.22 | 0.12 |
| 16-08-23 | 52.14 | 46.38 | 3.81 | 8.17 | 0.85 | 11.97 | 2.64 | 2.99 | 0.11 | 23.25 | 0.21 |
| 16-08-23 | 57.37 | 33.43 | 2.21 | 6.83 | 0.39 | 6.83 | 1.59 | 1.83 | 0.07 | 17.94 | 0.16 |
| 16-08-23 | 54.97 | 31.33 | 3.34 | 4.91 | 0.87 | 6.55 | 2.25 | 1.42 | 0.05 | 18.45 | 0.17 |
| 16-08-23 | 50.87 | 35.50 | 2.34 | 6.61 | 0.44 | 9.32 | 1.65 | 2.83 | 0.10 | 16.75 | 0.15 |
| 16-08-23 | 67.26 | 23.83 | 2.66 | 5.72 | 0.57 | 5.93 | 1.95 | 1.46 | 0.04 | 10.72 | 0.10 |
| 16-08-26 | 44.65 | 35.21 | 2.33 | 4.43 | 0.44 | 11.56 | 1.69 | 2.02 | 0.04 | 17.20 | 0.16 |
| 16-08-27 | 50.64 | 49.27 | 2.68 | 9.87 | 0.80 | 16.29 | 1.61 | 3.36 | 0.08 | 19.75 | 0.18 |
| 16-08-27 | 47.76 | 27.14 | 1.25 | 3.27 | 0.15 | 9.80 | 0.97 | 1.51 | 0.03 | 12.56 | 0.11 |
| 16-08-28 | 46.63 | 22.20 | 0.73 | 4.72 | 0.22 | 5.15 | 0.39 | 1.61 | 0.03 | 10.72 | 0.09 |
| **Mean** | **55.44** | **29.75** | **2.08** | **5.43** | **0.43** | **8.06** | **1.47** | **2.28** | **0.06** | **13.98** | **0.13** |
| **SD** | **20.49** | **10.40** | **0.94** | **3.06** | **0.24** | **4.19** | **0.75** | **1.66** | **0.04** | **4.45** | **0.04** |

Table S9: Zooplankton net sample species identification and enumeration data. Samples collected from oblique tows are in italics and the others were obtained from vertical hauls. Samples shaded in grey are from Pangnirtung Fiord and the unshaded samples were collected in Kingnait Fiord.

Total counted is the total number of organisms identified per aliquot per sample while the total sampled is the total number of zooplankton collected in each net tow. The total abundance is the number of organisms per cubic meter. Aliquot species counts are provided for the most numerous taxa.

| **Date** | **Total counted** | | **Total sampled** | | **Total abundance** | ***C. hyp*** | | ***C. glac*** | ***C. fin*** | ***Pseudo* spp.** | ***Metridia spp.*** | ***Oithona* spp.** | ***Acartia longiremis*** | **P*areuchaeta* spp.** | ***Cyclops* spp.** |
| --- | --- | --- | --- | --- | --- | --- | --- | --- | --- | --- | --- | --- | --- | --- | --- |
| 16-08-05 | | 209 | 1254 | 88.75 | | 9 | 21 | | 20 | 127 | 1 | 0 | 2 | 0 | 0 |
| 16-08-05 | | 373 | 1119 | 113.97 | | 4 | 38 | | 119 | 188 | 0 | 0 | 0 | 0 | 0 |
| 16-08-05 | | 387 | 1161 | 118.32 | | 4 | 30 | | 88 | 145 | 1 | 0 | 3 | 0 | 0 |
| 16-08-05 | | 448 | 1344 | 129.22 | | 5 | 51 | | 88 | 175 | 0 | 0 | 1 | 0 | 0 |
| 16-08-07 | | 347 | 1041 | 82.88 | | 40 | 90 | | 62 | 125 | 1 | 1 | 1 | 0 | 4 |
| 16-08-10 | | 263 | 1052 | 48.98 | | 26 | 64 | | 41 | 105 | 0 | 0 | 1 | 0 | 1 |
| 16-08-10 | | 280 | 1960 | 32.56 | | 44 | 33 | | 3 | 149 | 37 | 0 | 0 | 0 | 0 |
| 16-08-15 | | 237 | 1422 | 20.05 | | 25 | 65 | | 13 | 102 | 26 | 0 | 0 | 0 | 0 |
| *16-08-15* | | *212* | *1696* | *17.86* | | *37* | *29* | | *5* | *113* | *23* | *0* | *0* | *0* | *0* |
| 16-08-16 | | 212 | 1272 | 22.51 | | 44 | 27 | | 14 | 101 | 12 | 0 | 0 | 0 | 0 |
| *16-08-16* | | *239* | *956* | *33.93* | | *48* | *53* | | *35* | *62* | *1* | *0* | *0* | *0* | *0* |
| *16-08-16* | | *424* | *1696* | *61.49* | | *99* | *100* | | *39* | *128* | *1* | *0* | *1* | *0* | *1* |
| *16-08-16* | | *462* | *924* | *32.70* | | *94* | *76* | | *36* | *94* | *3* | *0* | *0* | *1* | *0* |
| 16-08-19 | | 263 | 1578 | 28.93 | | 0 | 97 | | 46 | 109 | 0 | 0 | 0 | 0 | 0 |
| 16-08-19 | | 323 | 2261 | 23.67 | | 51 | 42 | | 3 | 191 | 29 | 0 | 0 | 0 | 0 |
| 16-08-20 | | 200 | 1200 | 40.06 | | 13 | 76 | | 22 | 65 | 2 | 0 | 0 | 0 | 0 |
| 16-08-19 | | 440 | 2640 | 29.39 | | 74 | 102 | | 6 | 198 | 52 | 0 | 0 | 0 | 0 |
| 16-08-23 | | 422 | 2532 | 48.56 | | 71 | 104 | | 26 | 202 | 6 | 0 | 1 | 0 | 0 |
| 16-08-23 | | 328 | 2296 | 40.02 | | 56 | 56 | | 15 | 147 | 34 | 0 | 0 | 0 | 0 |
| 16-08-23 | | 332 | 1992 | 36.24 | | 45 | 60 | | 13 | 170 | 33 | 0 | 0 | 0 | 0 |
| 16-08-23 | | 327 | 1962 | 38.57 | | 56 | 79 | | 24 | 142 | 11 | 1 | 0 | 0 | 0 |
| 16-08-23 | | 311 | 2177 | 32.37 | | 55 | 57 | | 14 | 104 | 71 | 0 | 0 | 1 | 0 |
| 16-08-26 | | 284 | 1704 | 38.16 | | 33 | 86 | | 15 | 128 | 9 | 0 | 0 | 0 | 0 |
| 16-08-27 | | 532 | 2660 | 52.53 | | 100 | 165 | | 34 | 200 | 1 | 0 | 0 | 0 | 0 |
| 16-08-27 | | 228 | 1368 | 28.64 | | 26 | 78 | | 12 | 100 | 0 | 0 | 0 | 0 | 0 |
| 16-08-28 | | 227 | 1135 | 24.34 | | 44 | 48 | | 15 | 100 | 4 | 0 | 0 | 0 | 0 |

Table S10: Percent (% C*alanus* spp.) of all *Calanus* spp. organisms enumerated (n = 323) from bowhead whale stomach contents. Data are organized by species and life-stage.

|  | **Total** | **% C*alanus* spp.** |
| --- | --- | --- |
| ***Calanus hyperboreus*** |  |  |
| early | 85 | 26.3 |
| late | 8 | 2.5 |
| unstaged | 4 | 1.2 |
| ***Calanus glacialis*** |  |  |
| early | 26 | 8.0 |
| late | 79 | 24.5 |
| unstaged | 4 | 1.2 |
| ***Calanus finmarchicus*** |  |  |
| early | 0 | 0 |
| late | 4 | 1.2 |
| unstaged | 0 | 0 |
| ***Calanus* spp.** |  |  |
| unstaged | 113 | 35.0 |
